# Enzyme-Loaded pH-Sensitive Photothermal Hydrogels for Mild-temperature-mediated Combinational Cancer Therapy

**DOI:** 10.3389/fchem.2021.736468

**Published:** 2021-07-29

**Authors:** Jindong Xia, Xueqin Qing, Junjian Shen, Mengbin Ding, Yue Wang, Ningyue Yu, Jingchao Li, Xiuhui Wang

**Affiliations:** ^1^ Department of Radiology, Shanghai Songjiang District Central Hospital, Shanghai, China; ^2^ Department of Pediatrics, Shanghai General Hospital, Shanghai Jiao Tong University, School of Medicine, Shanghai, China; ^3^ Department of Radiology, The First Affiliated Hospital of Bengbu Medical College, Bengbu, China; ^4^ Shanghai Engineering Research Center of Nano-Biomaterials and Regenerative Medicine, College of Chemistry, Chemical Engineering and Biotechnology, Donghua University, Shanghai, China; ^5^ Institute of Translational Medicine, Shanghai University, Shanghai, China

**Keywords:** hydrogels, photothermal therapy, starvation therapy, second near-infrared light, tumor metastasis, cancer therapy

## Abstract

Photothermal therapy (PTT) that utilizes hyperthermia to ablate cancer cells is a promising approach for cancer therapy, while the generated high temperature may lead to damage of surrounding normal tissues and inflammation. We herein report the construction of glucose oxidase (GOx)-loaded hydrogels with a pH-sensitive photothermal conversion property for combinational cancer therapy at mild-temperature. The hydrogels (defined as CAG) were formed *via* coordination of alginate solution containing pH-sensitive charge-transfer nanoparticles (CTNs) as the second near-infrared (NIR-II) photothermal agents and GOx. In the tumor sites, GOx was gradually released from CAG to consume glucose for tumor starvation and aggravate acidity in tumor microenvironment that could turn on the NIR-II photothermal conversion property of CTNs. Meanwhile, the released GOx could suppress the expression of heat shock proteins to enable mild NIR-II PTT under 1,064 nm laser irradiation. As such, CAG mediated a combinational action of mild NIR-II PTT and starvation therapy, not only greatly inhibiting the growth of subcutaneously implanted tumors in a breast cancer murine model, but also completely preventing lung metastasis. This study thus provides an enzyme loaded hydrogel platform with a pH-sensitive photothermal effect for mild-temperature-mediated combinational cancer therapy.

## Introduction

Photothermal therapy (PTT) that utilizes photoconversion to produce heat for tumor ablation has been explored as a non-invasive therapeutic strategy for cancer (Cheng et al., 2017; Jung et al., 2018; Zhao et al., 2018). In view of the high spatiotemporal controllability of light, PTT has the advantages of high treatment specificity and minimal side effects, which is different from conventional therapeutic strategies such as radiotherapy and chemotherapy (Liu et al., 2011; Ju et al., 2015; Li and Pu, 2019; 2020). High temperature (>50°C) is usually required to induce complete tumor cell death during PTT, which potentially results in damage of surrounding normal tissues and inflammation (Zhu et al., 2016; Yang et al., 2017; Gao et al., 2019). Therefore, mild PTT strategies at a low therapeutic temperature have attracted a great attention (Zhou et al., 2018; Ding et al., 2020; Yuan et al., 2020). However, the therapeutic efficacy of mild PTT is often compromised by the upregulated expression of heat shock proteins (HSPs) that are associated with hyperthermia-induced cell damage (Ali et al., 2016; Wang et al., 2016; Wang et al., 2017). To overcome heat resistance of cancer cells, HSP inhibitors have been used to amplify the effect of mild PTT (Tang et al., 2018). Most of existing mild PTT strategies are relied on the first near-infrared (NIR) light (NIR-I, 650–950 nm) that shows too shallow tissue penetration depth to deliver sufficient heating to the internal regions of solid tumors (Gao et al., 2021). Compared to NIR-I light, the second near-infrared (NIR-II) light (1,000–1700 nm) has greatly improved penetrating capability in biological tissues (Li et al., 2021; Luo et al., 2021; Wang et al., 2021). In this regard, it is highly desired to develop mild NIR-II PTT for tumor ablation with high safety and efficacy.

Starvation therapy that blocks the energy metabolism of cancer cells has emerged as an effective therapeutic strategy for cancer (Guo and Kohane, 2017; Zhang et al., 2017; Zhang et al., 2019). To date, some strategies such as vascular embolization, inhibition of glucose transporter, and direct intratumoral glucose consumption have been adopted to starve cancer cells (Butler et al., 2013; Liu et al., 2017; Cheng et al., 2019; Tang et al., 2021). Among them, glucose oxidase (GOx)-based starvation therapy *via* catalyzing the oxidation of glucose in tumor cells has achieved remarkable efficacy in inhibiting tumor growth (Dinda et al., 2018; Fu et al., 2018; Ranji-Burachaloo et al., 2019). However, this therapeutic model often encounters the issues of low therapeutic benefits and potential systemic toxicity (Ren et al., 2020). In addition, GOx-mediated tumor starvation can downregulate the expressions of HSPs due to the blocking of energy supply, which will contribute to enhanced PTT efficacy (Wang et al., 2012; Chen et al., 2017; Cao et al., 2020). The combination of PTT and starvation therapy has been adopted to treat tumors, which indeed achieves high antitumor efficacy with the neglect of side effects (Hu et al., 2019; Gao et al., 2020; Wang et al., 2020; He et al., 2021). Therefore, it is still highly desired to explore new strategies that integrate PTT and starvation therapy with combinational action for cancer treatment with high efficacy and safety.

**FIGURE 1:**
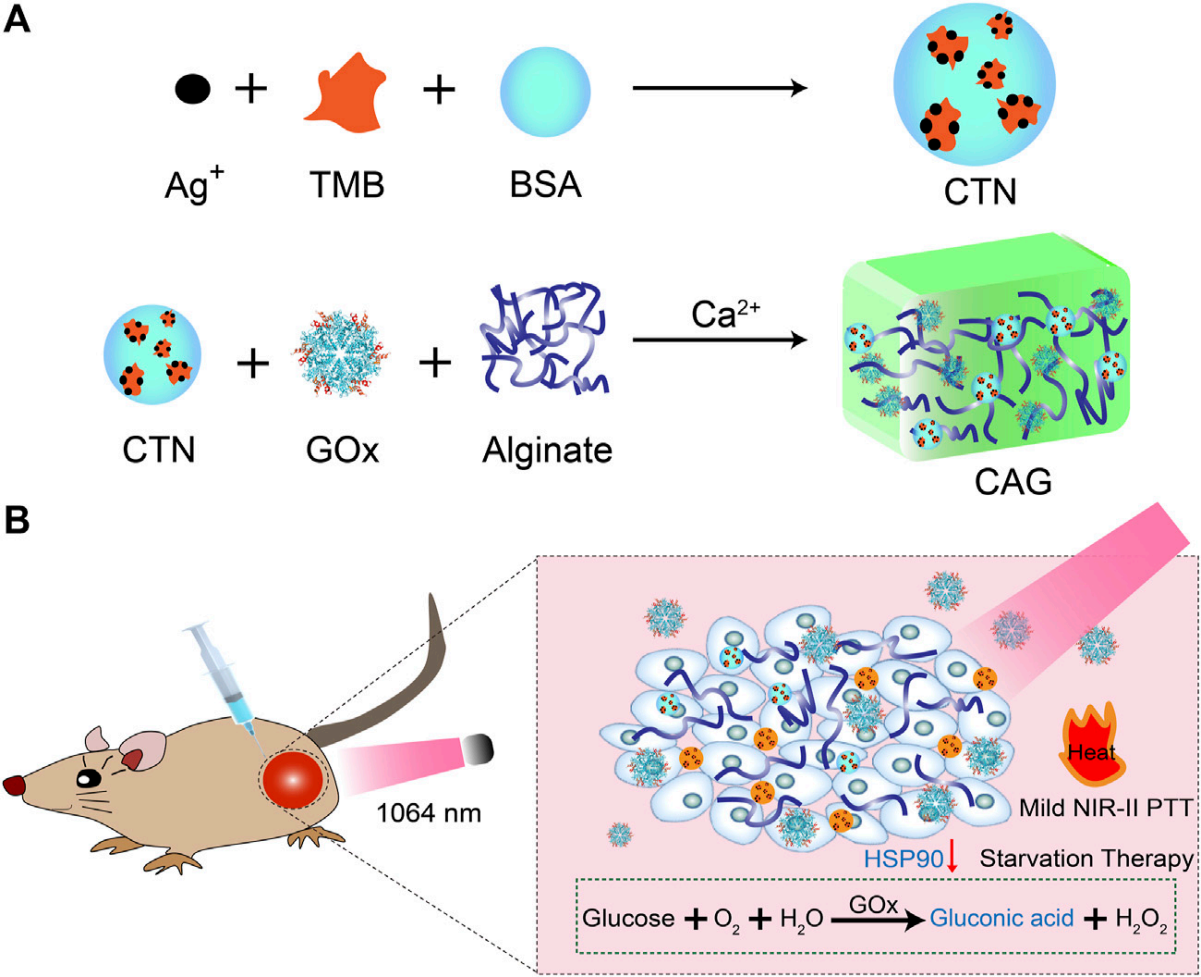
Design and fabrication of CAG hydrogels for mild-temperature-mediated NIR-II PTT and starvation combinational therapy. **(A)** Schematic illustration of the preparation of CAG hydrogels. **(B)** Schematic illustration of working mechanism of CAG hydrogels for mild-temperature-mediated NIR-II PTT and starvation combinational therapy.

In this study, we report a GOx-loaded alginate hydrogel with pH-sensitive NIR-II photothermal effect for treatment of solid tumors *via* combinational action at mild-temperature (Figure 1A). Alginate is used as matrix to construct hydrogels because of its excellent biocompatibility and degradability (Lee and Mooney, 2012). Alginate hydrogels have unique properties of good gelling capacity, low toxicity, excellent injectability, and low cost, and thus have been used for drug delivery, cancer therapy, molecular imaging and tissue engineering (Ouyang et al., 2019; Hernández-González et al., 2020; Johnson et al., 2020; Patrick et al., 2020). The CAG hydrogels containing pH-sensitive charge-transfer nanoparticles (CTNs) as the NIR-II photothermal agents and GOx as the starvation therapeutic agents can be locally formed *via* Ca^2+^ coordination in tumor tissues (Wang et al., 2019; Liu et al., 2021). Glucose was consumed to enable starvation therapy because of gradual release of GOx from CAG hydrogels, which also led to aggravated acidity in tumor microenvironment and inhibited expression of HSP90. As such, NIR-II PTT effect of CTNs was activated to mediate effective tumor ablation at a low temperature. Therefore, CAG-mediated combinational action of mild NIR-II PTT and starvation therapy afforded much higher therapeutic efficacy relative to sole treatment (Figure 1B). Such treatment could not only significantly suppress the growth of subcutaneous 4T1 tumors in living mice, but also completely prevent lung metastasis.

## Materials and Methods

### Materials and Reagents

Bovine serum albumin (BSA), 3,3′,5,5′-tetramethylbenzidine (TMB), silver nitrate (AgNO_3_) and GOx were purchased from Sigma-Aldrich (St. Louis, United States). Anhydrous CaCl_2_ and sodium alginate were purchased from Aladdin Reagent Co. Ltd. (Shanghai, China). Cell counting kit-8 (CCK-8) and calcein-AM and propidium iodide apoptosis detection kit were purchased from Dojindo Laboratories (Kumamoto, Japan) and Dalian Meilun Biotech Co. Ltd. (Dalian, China), respectively. RPMI 1640 cell culture medium, fetal bovine serum (FBS), and penicillin-streptomycin were obtained from Gibco (Grand Island, NY, United States). TdT-mediated dUTP-biotin nick end labeling (TUNEL) kit was obtained from Roche (Sweden). Ultrapure water used in this study was prepared *via* a water purification system (PALL Cascada, MI, United States).

### Synthesis of Charge-Transfer Nanoparticles

To synthesize pH-sensitive CTNs, 4.8 mg TMB dissolved in anhydrous ethanol was added into 2 ml solution containing 1.7 mg AgNO_3_ and 3.4 mg BSA under ultrasonic oscillation at room temperature for 30 min. The products were purified by dialysis using a dialysis bag (molecular weight cut-off = 3 kDa) for 4 days to obtain CTNs.

### Synthesis of CAG Hydrogels

To synthesize CAG hydrogels, 1 mg GOx and 200 mg sodium alginate were co-dissolved in 15 ml phosphate buffer saline (PBS) and then mixed with CTNs at a final concentration of 20 mg/ml. The mixed solution was then injected into 12 ml Ca^2+^ solution (1.8 mM) in a crystal bottle, forming CAG hydrogels. Similarly, CA hydrogels without GOx loading were synthesized *via* injecting CTN solution into 12 ml Ca^2+^ solution (1.8 mM) and the formed CA hydrogels were used as control.

### Characterization Techniques

Dynamic light scattering (DLS) and zeta potential measurements of CTNs were used a Zetasizer Nano-series (Nano-ZS90, Malvern, United Kingdom). UV-vis-NIR absorption spectra of CTNs at different pH conditions were recorded on a Persee spectrophotometer (TU-1810, Beijing, China). Scanning electron microscopy (SEM) images of formed hydrogels were observed using a SEM (SU8010, HITACHI, Tokyo, Japan).

### Photothermal Effect Evaluation

The photothermal properties of CA and CAG hydrogels were evaluated by exposing samples under 1,064 nm laser at the power density of 1 W/cm^2^ for different time. In a typical experiment, the mixture of 20 μl CTN (0.96 mg/ml), 50 μL alginate (10 mg/ml) and 1 μl GOx (1 mg/ml) was added in a 96-well plate containing 30 μl Ca^2+^ aqueous solution. The temperature of mixed solution under laser irradiation was recorded using a Fotric 220s photothermal camera. Furthermore, the photothermal stability of hydrogels was investigated by turning on/off the laser for five cycles.

### Evaluation of Glucose Oxidase Release From Hydrogels

CAG hydrogels were prepared as above described, and the formed CAG hydrogels were put in 5 ml PBS solution under shaking at 37°C. After incubation for different time, supernatant was collected and then centrifugated for absorption measurement to confirm the release of GOx.

### *In vitro* Cell Apoptosis Analysis

To evaluate the *in vitro* therapeutic efficacy of hydrogels, cell apoptosis analysis was conducted. 4T1 cancer cells were seeded in 6-well plates (1 × 10^5^ cells/well) and incubated at 37°C for 24 h. Then the cells were treated with PBS, CA, or CAG hydrogels for 24 h, followed by 1,064 nm laser irradiation at the power density of 1 W/cm^2^ for 15 min. After culture for 12 h, the cells were incubated in cell culture medium containing calcein-AM/PI mixed solution for another 30 min. Fluorescence images of stained cells were captured using a fluorescence microscope (Leica DMi8, Germany). The green and red fluorescence area ratios were quantified using ImageJ software. For CCK-8 assay, 4T1 cancer cells were seeded in 96-well plates (1 × 10^4^ cells/well) and incubated at 37°C for 24 h. Then the cells were treated with PBS, CA, or CAG at different CTN concentration for 24 h. The treated cells were irradiated by 1,064 nm laser (1 W/cm^2^) for 5 min. The cells without laser irradiation were used as the control. After laser irradiation, the cells were cultured for 12 h, and then the cell culture medium was carefully removed and fresh medium containing CCK-8 agent was added into each well. After culture for another 2 h, the absorbance of each well at 450 nm was measured using a Bio-Tek ELX800 spectrophotometric microplate reader (Vermont, America). The absorbance was used to calculate the cell viability.

### *In vivo* Evaluation of HSP90 Expression During Photothermal Therapy

The animal procedures were approved by the Animal Care and Use Committee of Donghua University. Male 4–6 week-old BALB/c nude mice (∼20 g) were purchased from Shanghai SLAC Laboratory Animal Co., Ltd. 4T1 tumor-bearing mice were established by subcutaneously injecting 4T1 cancer cells (2 × 10^6^ cells/mouse) into the right flank of each mouse. The 4T1 tumor-bearing nude mice were randomly divided into six groups when the tumor volume reached ∼100 mm^3^. The tumors were treated with PBS, CA, or CAG for 12 h, and then exposed under 1,064 nm laser irradiation (1 W/cm^2^) for 10 min in a discontinuous manner. The temperature of tumor sites during laser irradiation was monitored using an IR thermal camera and controlled to be lower than 45°C. After treatment for 1 day, the mice were euthanized, and tumors were collected and used for immunofluorescent staining of HSP90. The fluorescence staining images of tumor sections were captured using a fluorescence microscope (Leica DMi8, Germany). The mean fluorescence intensity (MFI) of HSP90 staining was quantified using the ImageJ software.

### *In vivo* Antitumor Efficacy Evaluation

The 4T1 tumor-bearing nude mice were treated with PBS, CA, or CAG without or with 1,064 nm laser irradiation (1 W/cm^2^) for 10 min in a discontinuous manner to control tumor temperature below 45°C. After different treatments, a caliper was used to measure the tumor sizes every 2 days for 20 days. Tumor volumes were calculated as follows: volume = (length) × (width)^2^/2, and relative tumor volume was calculated as V/V_0_ (V_0_ was the initial tumor volume). After treatments for 20 days, the mice were euthanized, and the tumors were extracted and weighed to evaluate tumor inhibition ratios. The tumors were collected for hematoxylin and eosin (H&E) and immunohistochemical TUNEL and Ki67 staining.

### *In vivo* Anti-metastasis Efficacy Evaluation

After different treatments for 20 days, *in vivo* anti-metastasis efficacy was evaluated. The treated mice were intraperitoneally injected with 0.15 ml D-luciferin (20 mg/ml) and the peritoneum was opened to expose lungs. The lungs were then used for bioluminescence imaging using *in vivo* imaging system (VISQUE Invivo Smart-LF, Vieworks, Korea). Bioluminescence intensities of lungs were quantified using a Living Image software. To further assess the anti-metastasis efficacy of different treatments, the lungs were collected and washed with PBS, and the numbers of metastatic tumor nodes were counted. The collected lungs were then used for H&E staining to observe tumor metastasis.

### *In vivo* Biocompatibility Evaluation

After different treatments, the body weights of 4T1 tumor-bearing nude mice were measured every 2 days for 20 days to evaluate the *in vivo* biocompatibility. After treatments for 20 days, the mice were euthanized and heart, liver, spleen, and kidney were collected and used for H&E staining.

### Statistical Analysis

The significant difference between the experimental statistics is analyzed by One-way ANOVA and Tukey’s multiple comparison tests. When the *p*-values were <0.05, the values were statistically regarded to be significantly different. *p* < 0.05 was indicated by (*), *p* < 0.01 by (**) and *p* < 0.001 by (***).

## Results and Discussion

### Synthesis and Characterization of Hydrogels

**FIGURE 2:**
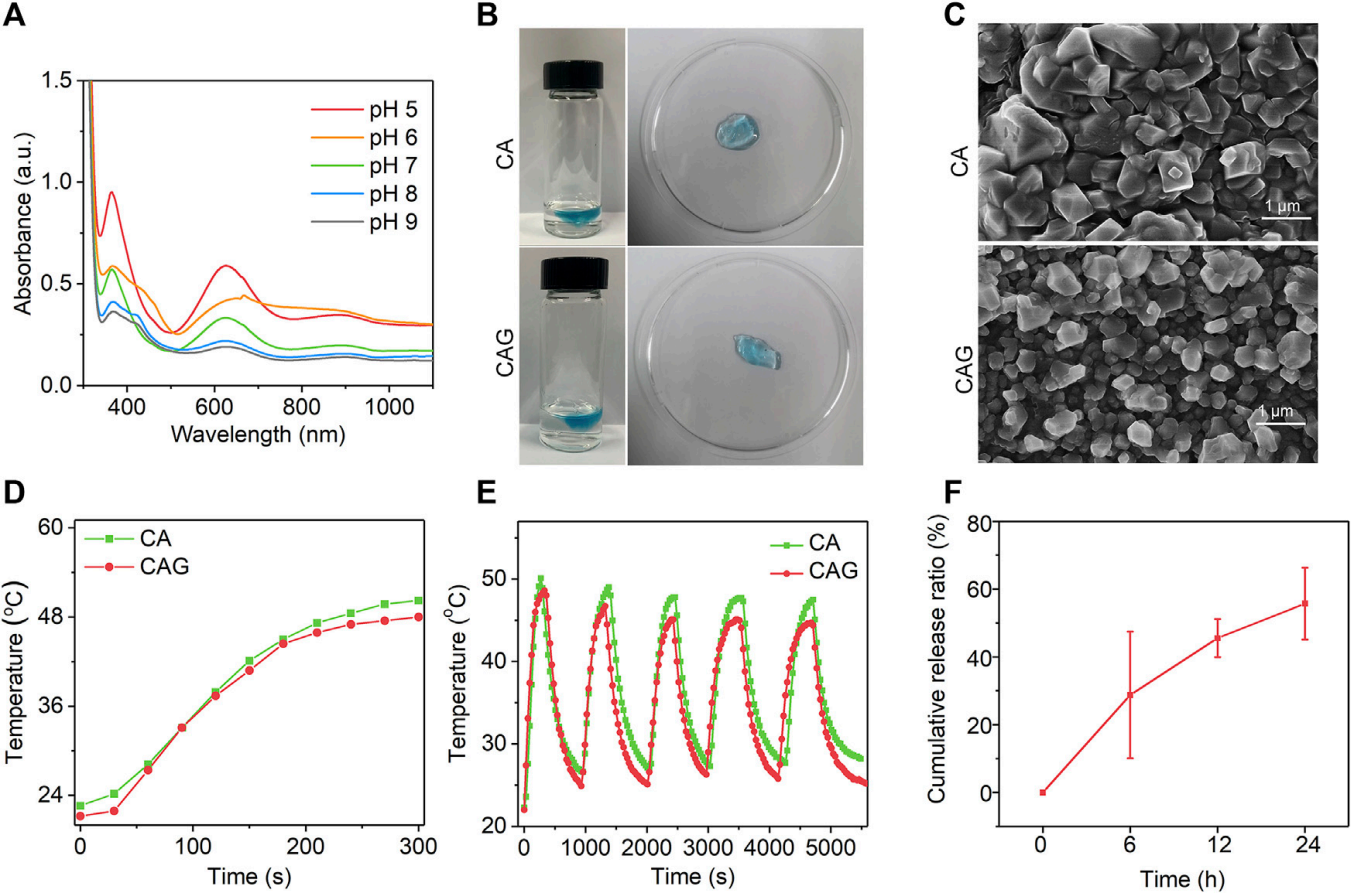
Characterization of CAG hydrogels. **(A)** UV-vis absorption spectra of CTN at different pH conditions. **(B)** Representative SEM images of CA and CAG hydrogels. **(C)** Photographs of CA and CAG hydrogels after injecting alginate solution (5 mg/ml) containing CTN or CTN and GOx into aqueous solution at the Ca^2+^ concentration of 1.8 mM. **(D)** Temperature changes of aqueous solution containing CA or CAG under 1,064 nm laser irradiation at the powder density of 1 W/cm^2^ for different time. **(E)** Evaluation of photothermal stability of CA and CAG hydrogels after five cycles of laser on/off. **(F)** Release profile of GOx from CAG hydrogels after incubation at 37°C for different time.

To construct GOx-loaded pH-sensitive photothermal hydrogels, pH-sensitive CTNs with activatable NIR-II photothermal conversion property were first synthesized. Hydrodynamic diameter of CTNs was measured to be 14.7 nm (Supplementary Figure S1, Supporting information). The surface zeta potential of CTNs was around -22.2 mV (Supplementary Figure S2, Supporting information). The characteristic absorption of CTNs at different pH conditions was different and higher absorption in the NIR-II regions could be observed at acidic conditions (Figure 2A). The color of CTN solution gradually changed from blue to gray as the increase of pH from 5 to 9 (Supplementary Figure S3, Supporting information). The photothermal property of CTN solutions was different at pH = 5, 7, or 9 under 1,064 nm laser irradiation. The temperature rise of CTNs at pH = 5 was much faster than those at pH = 7 and 9 (Supplementary Figure S4, Supporting information). These results indicated that the synthesized CTNs were pH-sensitive photothermal agents. The pH-responsive photothermal property of CTNs may be due to different charge-transfer efficiency between the components within nanoparticles at different pH conditions (Wang et al., 2019).

As shown in Figure 2B, CA hydrogels with loading of CTNs and CAG hydrogels with loadings of CTNs and GOx could be formed *via* coordination reaction of alginate with Ca^2+^. SEM images showed that the morphologies of CA and CAG were similar (Figure 2C), indicating that loading of CTNs and GOx did not affect the morphology of hydrogels. The photothermal performances of CA and CAG hydrogels were evaluated under 1,064 nm laser irradiation. Heating curves and thermal images showed that the temperatures increased rapidly for solutions containing CA and CAG hydrogels under 1,064 nm laser irradiation at the power density of 1 W/cm^2^ for 5 min (Figure 2D and Supplementary Figure S5, Supporting information).There was no significant difference in the aspect of photothermal property between CA and CAG hydrogels, suggesting the loading of GOx showed neglectful influence on the photothermal effect of hydrogels. Moreover, the temperature increase of hydrogels did not have obvious changes after five cycles of laser on/off, indicating that CA and CAG hydrogels had good photothermal stability (Figure 2E). The release profile showed that GOx was gradually released form CAG hydrogels, and the cumulative release ratio could reach 76.0% after incubation at 37°C for 24 h (Figure 2F).

### Evaluation of *in vitro* Therapeutic Efficacy to 4T1 Cancer Cells

**FIGURE 3:**
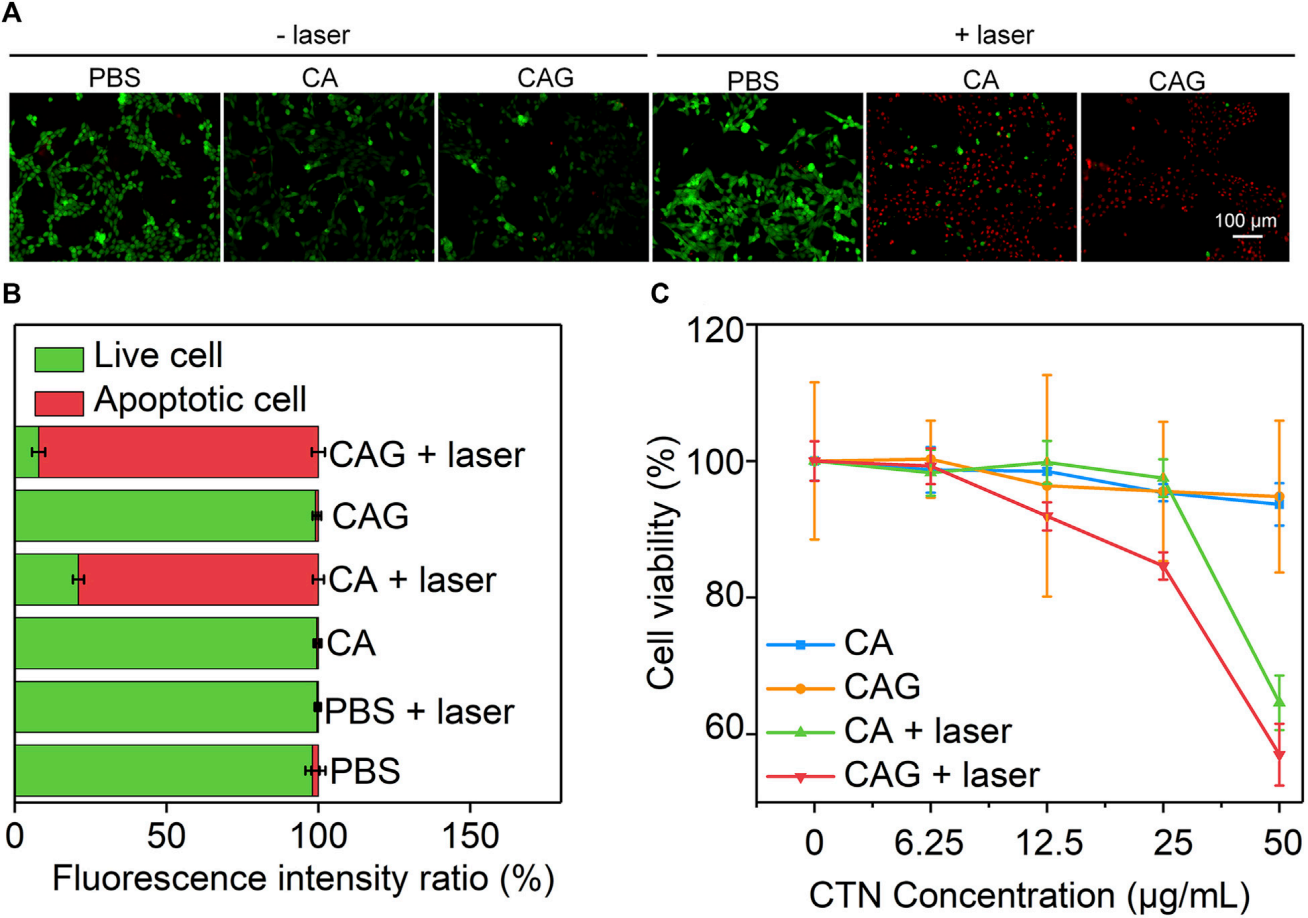
Evaluation of *in vitro* therapeutic efficacy of hydrogels. **(A)** Fluorescence images of live (green) and dead (red) 4T1 cancer cells after incubation with PBS, CA or CAG hydrogels without or with 1,064 nm laser irradiation (1 W/cm^2^) for 15 min. **(B)** Quantification of fluorescence intensity of 4T1 cancer cells after different treatments. **(C)** Cell viability of 4T1 cancer cells after incubation with PBS, CA or CAG hydrogels at different CTN concentrations without or with 1,064 nm laser irradiation (1 W/cm^2^) for 5 min.

*In vitro* therapeutic efficacy of CA and CAG hydrogels was evaluated using 4T1 cancer cells. Fluorescence images showed that obvious dead cells (red fluorescence signals) were observed in CA and CAG groups after 1,064 nm laser irradiation at the power density of 1 W/cm^2^ for 15 min, while almost no dead cells were found in control and the other treatment groups (Figure 3A). The quantitative assay of fluorescence intensity indicated that the percentage of apoptotic cells was 79.0 and 97.8% for CA and CAG treatment and laser irradiation, respectively, while the percentage of apoptotic cells was less than 2% in the other groups (Figure 3B). Moreover, the therapeutic efficacy was evaluated by measuring the cell viability of 4T1 cancer cells after different treatments. Without laser irradiation, the cell viability of both CA and CAG treated cells at different CTN concentrations was higher than 90%, which indicated there was no obvious cytotoxicity for hydrogels. However, after 1,064 nm laser irradiation at the power density of 1 W/cm^2^ for 5 min, the cell viability was decreased with the increase of CTN concentrations (Figure 3C). At the same CTN concentrations, the cell viability of 4T1 cancer cells after CAG treatment plus laser irradiation was lower relative to that after CA treatment with laser irradiation. These results suggested that CA and CAG could kill cancer cells *via* PTT effect, and the therapeutic efficacy of CAG was higher than that of CA.

### Evaluation of HSP90 Expression During Photothermal Therapy

**FIGURE 4:**
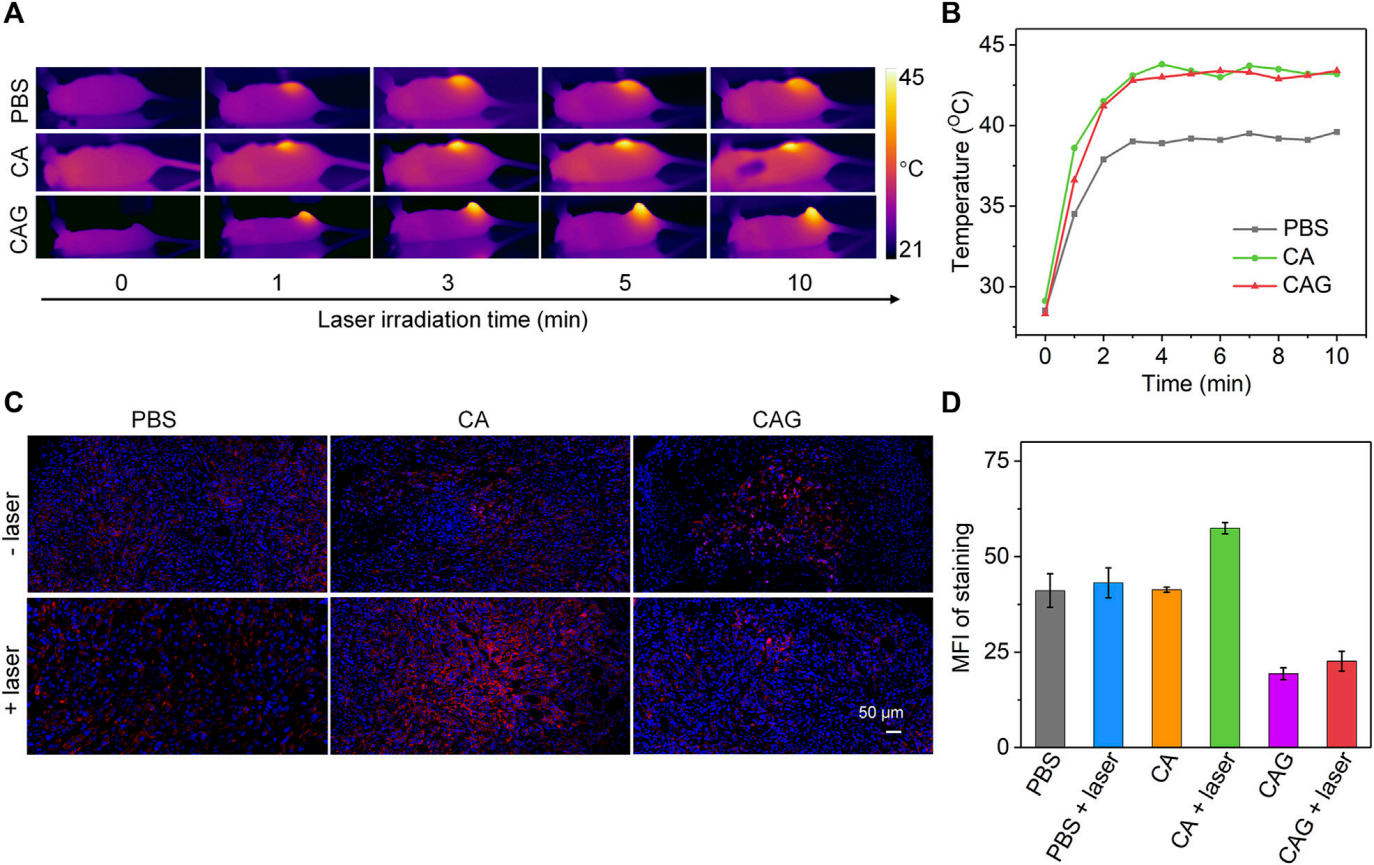
Evaluation of mild NIR-II PTT and intratumor expression of HSP90. **(A)** *In vivo* Thermal imaging of 4T1 tumor-bearing mice after treatment with PBS, CA, or CAG hydrogels under 1,064 nm laser irradiation at the powder density of 1 W/cm^2^ for 10 min. **(B)** Temperature changes of tumor sites of 4T1 tumor-bearing mice after different treatments under 1,064 nm laser irradiation (1 W/cm^2^) for different time. **(C)** Immunofluorescence HSP90 staining of tumors after different treatments. The blue fluorescence signals indicated cell nucleus stained by 4′,6-diamidino-2-phenylindole (DAPI), and the red fluorescence signals indicated HSP90 stained by antibody. **(D)** Mean fluorescence intensity (MFI) of HSP90 staining in different groups.

4T1 tumor-bearing mice were used as models to investigate the therapeutic efficacy of hydrogels. To achieve mild NIR-II PTT and starvation combinational therapy, the temperature of tumors during PTT should be controlled below 45°C. After treatment with PBS, CA or CAG, tumors were irradiated with 1,064 nm laser in a discontinuous manner and the tumor temperatures were monitored. During laser irradiation, the temperatures of tumor sites for CA and CAG treated mice gradually increased, and similarly reached around 44°C after 3 min of laser irradiation and maintained at this temperature for another 7 min (Figures 4A,B). The tumor temperature for mice after treatment with PBS only reached around 39°C after 10 min of laser irradiation.

The expressions of HSP90 in tumor tissues after different treatments were then evaluated. As shown in the immunofluorescence staining images, the strongest staining signal was detected for the tumors after treatment with CA plus 1,064 nm laser irradiation, suggesting PTT upregulated the expression of HSP90 (Figure 4C). Compared to the PBS control group, the staining signals for CAG treated tumors regardless of laser irradiation were much weaker. Quantitative assay showed that the tumors after CA treatment plus laser irradiation had the highest expression level of HSP90, while the expression level of HSP90 in tumors after CAG treatment plus laser irradiation was reduced (Figure 4D). This indicated that CAG treatment could greatly inhibit the expression of HSP90 due to gradual release of GOx from hydrogels.

### *In vivo* Antitumor Efficacy Evaluation

**FIGURE 5:**
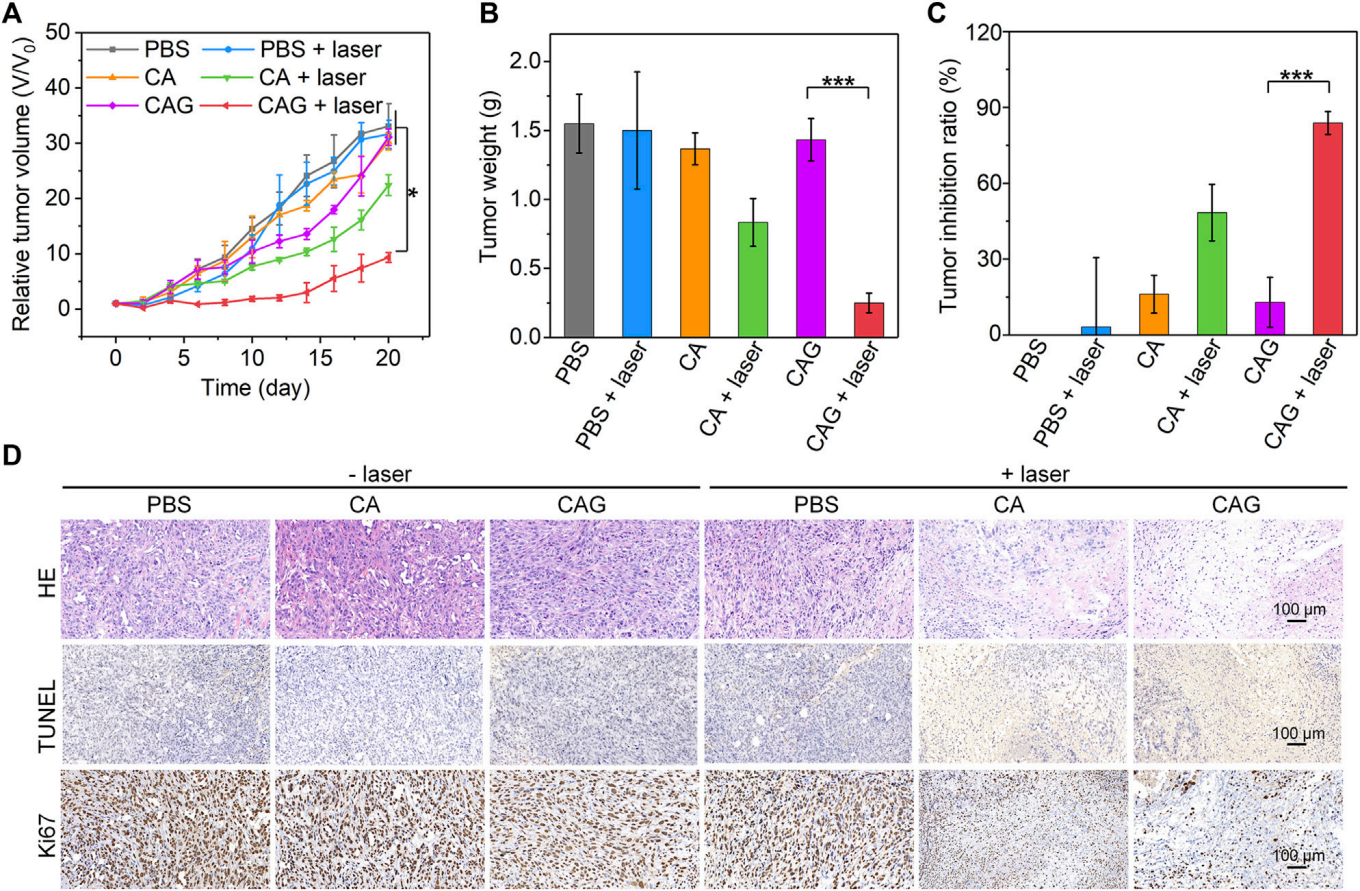
Evaluation of *in vivo* antitumor efficacy of hydrogels. **(A)** Relative tumor volumes of 4T1 tumor-bearing mice after treatment with PBS, CA, or CAG hydrogels under 1,064 nm laser irradiation at the powder density of 1 W/cm^2^ for 10 min. **(B)** Tumor weights of 4T1 tumors of mice after different treatments. **(C)** Tumor inhibition ratio of 4T1 tumors in tumor-bearing mice after different treatments. **(D)** H&E, TUNEL and Ki67 staining of 4T1 tumors from mice after different treatments.

To evaluate the antitumor efficacy of hydrogels, 4T1 tumor-bearing mice were treated with PBS, CA, or CAG hydrogels, followed by 1,064 nm laser irradiation for 10 min in a discontinuous manner to maintain the maximum tumor temperature below 45°C. Compared to the PBS control group, only the growths of tumors from SA and SAC treated mice with laser irradiation were inhibited, suggesting the effective therapeutic efficacy (Figure 5A). The slight inhibition of tumor growth for CA treated and laser irradiated mice should be due to sole mild NIR-II PTT. The relative tumor volume in the SAC treated and laser irradiated mice were much lower than that for the mice after treatment with CA plus laser irradiation. Such a higher therapeutic efficacy for CAG was attributed to the combinational action of mild NIR-II PTT and starvation therapy. Tumor weights in CA and CAG-mediated treatment groups were lower than those in the other groups (Figure 5B). In particular, the tumor weight for CAG treated and laser irradiated mice were 5.7-fold lower relative to that for CAG treated mice without laser irradiation. The tumor inhibition rate for CAG treatment plus laser irradiation was calculated to be 83.0%, which was 1.7 and 6.4-fold higher than that for CA plus laser treatment and sole CAG treatment, respectively (Figure 5C).

To further investigate the therapeutic efficacies of hydrogels, histological staining of tumors was performed. As shown in H&E staining images, necrotic tumor cells were clearly observed in the CA and CAG treated and laser irradiated groups, while which were almost not found in tumors after the other treatment (Figure 5D). The necrosis in CAG treated and laser irradiated tumors was much more conspicuous than that in CA treated and laser irradiated tumors. The results of immunohistochemical TUNEL staining also indicated that obvious staining of necrotic cells could be found in CA and CAG treated tumors with laser irradiation, while nearly no cell necrosis was observed in the other treatment groups. The staining signal of necrotic cells in CAG treated and laser irradiated group was stronger than that in CA treated and laser irradiated group. Furthermore, the tumors after treatment with CA and CAG plus laser irradiation showed lower expressions of Ki67 as compared to the control group, indicating CA and Cag-mediated therapy could inhibit the proliferation of tumor cells. The inhibitory efficacy for CAG-mediated therapy was higher than that of CA-mediated treatment. The histological staining results were consistent with the tumor growth results, further confirming that CAG exhibited higher antitumor efficacy than CA.

The body weights of 4T1 tumor-bearing mice after different treatments for 20 days were almost the same as that of control mice (Supplementary Figure S6, Supporting information). H&E staining images of heart, liver, spleen and kidney from 4T1 tumor-bearing mice showed that no abnormal morphologies were found for these tissues after CA and CAG treatments plus 1,064 nm laser irradiation (Supplementary Figure S7, Supporting information). These results demonstrated that CA and CAG-mediated therapy did not cause obvious systematic toxicity.

### *In vivo* Anti-metastasis Efficacy Evaluation

**FIGURE 6:**
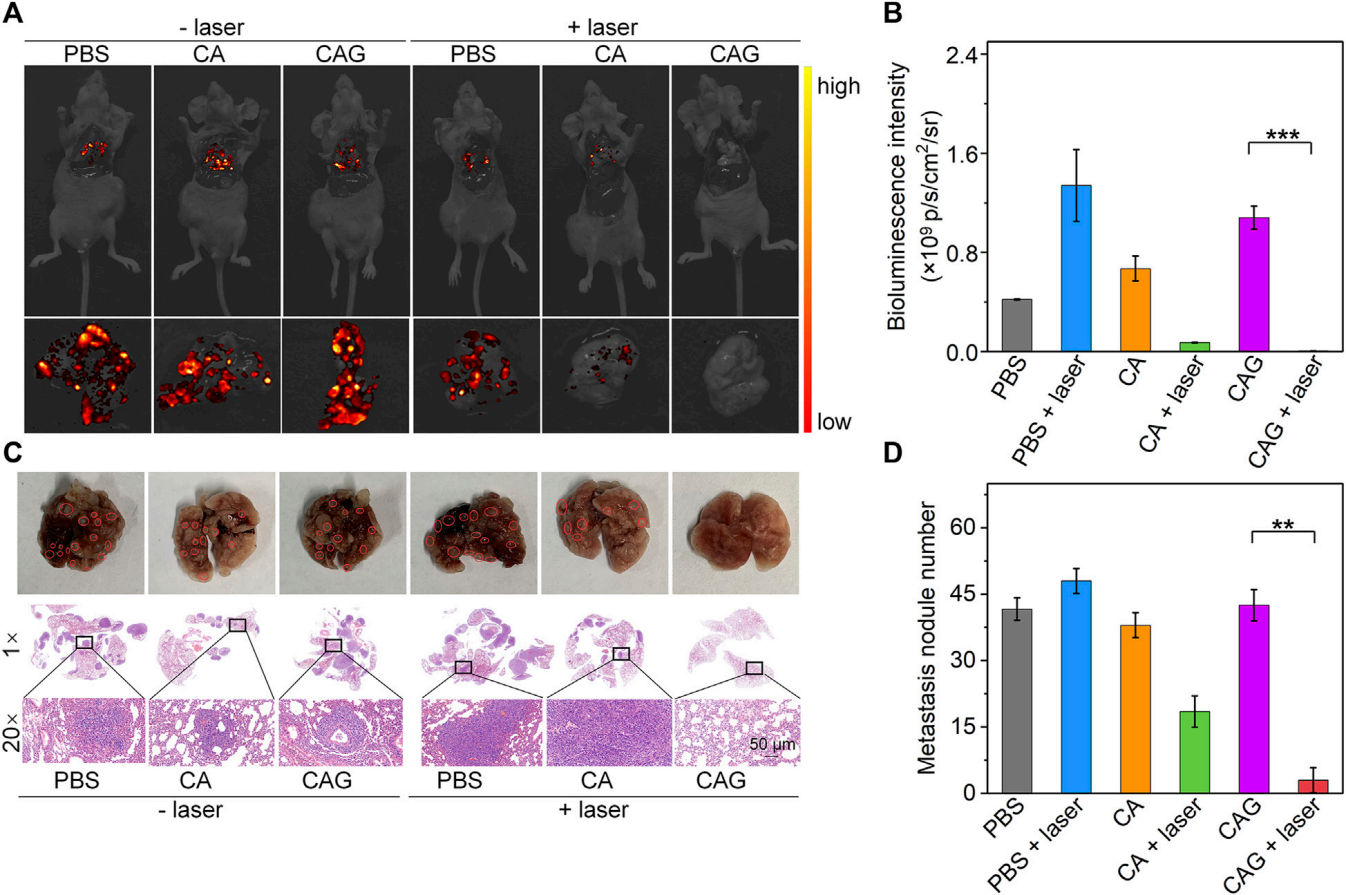
Evaluation of *in vivo* anti-metastasis efficacy of hydrogels. **(A)** Bioluminescence images of lung from 4T1 tumor-bearing mice after treatment with PBS, CA, or CAG hydrogels under 1,064 nm laser irradiation at the powder density of 1 W/cm^2^ for 10 min. **(B)** Bioluminescence intensity of lung from 4T1 tumor-bearing mice after different treatments. **(C)** Photographs and H&E staining images of lung from mice after different treatments. Red circles indicate the metastatic tumor nodes. **(D)** Number of metastatic tumor nodes of lungs from mice after different treatments.

In addition to inhibition of tumor growth, prevention of tumor metastasis is necessary to achieve ideal treatment of tumors. Bioluminescence imaging was conducted to evaluate the anti-metastasis efficacy of hydrogels. Obvious bioluminescence signals were observed in lungs of mice after treatments with PBS, CA, or CAG without 1,064 nm laser irradiation and mice treated with PBS plus laser irradiation (Figure 6A). The bioluminescence signals in lungs of CA or CAG hydrogel-treated and laser irradiated mice were much lower than those in the other groups. More importantly, nearly no bioluminescence signal could be detected for mice after treatment with CAG hydrogels with 1,064 nm laser irradiation. The quantitative analysis showed that the bioluminescence intensity for CA or CAG treated mice with laser irradiation was much lower relative to those for the other treated mice (Figure 6B). The lowest bioluminescence intensity was found in lungs of mice after CAG treatment plus laser irradiation.

H&E staining was also used to evaluate the lung metastasis after different treatments. As shown in the photographs and H&E staining images, metastatic tumor nodes were not observed in the lungs of mice after treatment with CAG hydrogels plus laser irradiation, which however were clearly observed in lungs of mice in the other treated groups (Figure 6C). The treatment of CA or CAG hydrogels plus laser irradiation greatly reduced the numbers of metastatic tumor nodes as compared to the treatments of PBS, CA, or CAG without laser irradiation and PBS plus laser irradiation (Figure 6D). In particular, the number of tumor metastasis in lungs of CAG treated and laser irradiated mice was significantly lower than that in lungs of CAG treated mice without laser irradiation. These results suggested that CAG-mediated therapy greatly prevented lung metastasis of 4T1 tumors.

## Conclusion

We have constructed a GOx-loaded smart hydrogel with pH-sensitive photothermal conversion property for combinational NIR-II PTT and starvation therapy of solid tumors at mild-temperature. The hydrogels (CAG) were locally formed after intratumoral injection of alginate solution containing CTN and GOx, which enabled gradual release of GOx into tumor sites. Through consuming glucose, CAG mediated starvation therapy, which not only led to exhaustion of tumor cells, but also resulted in aggravated acidity in tumor microenvironment and downregulated expression of HSP90. The NIR-II photothermal conversion property of CTNs was activated in acidic condition, which allowed for mild NIR-II PTT with a high efficacy due to the inhibited expression of HSP90. *Via* the combinational action of mild MIR-II PTT and starvation therapy, CAG was able to greatly suppress the growth of subcutaneously implanted tumors and completely prevent lung metastasis in a breast cancer murine model, while sole mild MIR-II PTT failed to do so. To the best of our knowledge, this study reports the first smart hydrogel platform with pH-sensitive NIR-II photothermal effect for mild-temperature-mediated combinational cancer therapy.

## Data Availability

The original contributions presented in the study are included in the article/Supplementary Files, further inquiries can be directed to the corresponding authors.
