# Supplementary figures and images for: Enzyme-Loaded pH-Sensitive Photothermal Hydrogels for Mild-temperature-mediated Combinational Cancer Therapy (part 2 of 2)
Source: Front Chem. 2021 Jul 29;9:736468. doi: 10.3389/fchem.2021.736468 (PMC8358069; doi:10.3389/fchem.2021.736468)

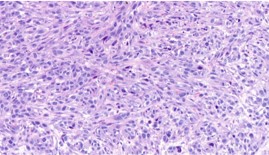

Supplement: Supplementary file 1 [file DataSheet1.ZIP › Original data/staining/HE/CAG.jpg]

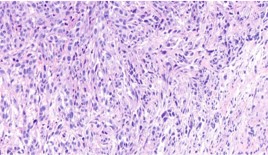

Supplement: Supplementary file 1 [file DataSheet1.ZIP › Original data/staining/HE/PBS+L.jpg]

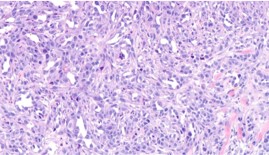

Supplement: Supplementary file 1 [file DataSheet1.ZIP › Original data/staining/HE/PBS.jpg]

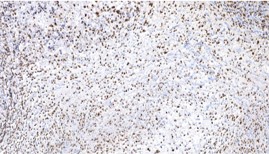

Supplement: Supplementary file 1 [file DataSheet1.ZIP › Original data/staining/KI67/CA+L.jpg]

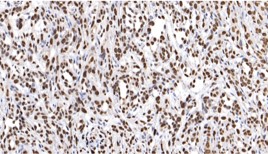

Supplement: Supplementary file 1 [file DataSheet1.ZIP › Original data/staining/KI67/CA.jpg]

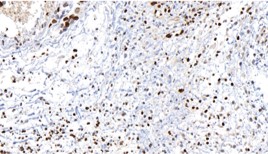

Supplement: Supplementary file 1 [file DataSheet1.ZIP › Original data/staining/KI67/CAG+L.jpg]

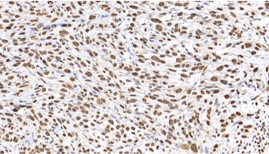

Supplement: Supplementary file 1 [file DataSheet1.ZIP › Original data/staining/KI67/CAG.jpg]

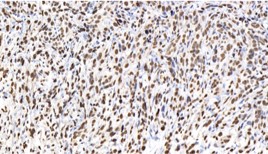

Supplement: Supplementary file 1 [file DataSheet1.ZIP › Original data/staining/KI67/PBS+L.jpg]

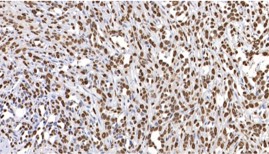

Supplement: Supplementary file 1 [file DataSheet1.ZIP › Original data/staining/KI67/PBS.jpg]

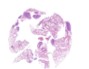

Supplement: Supplementary file 1 [file DataSheet1.ZIP › Original data/staining/Lung-HE/CA+L╖╬_1.0x.jpg]

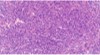

Supplement: Supplementary file 1 [file DataSheet1.ZIP › Original data/staining/Lung-HE/CA+L╖╬_20.0x.jpg]

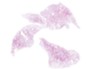

Supplement: Supplementary file 1 [file DataSheet1.ZIP › Original data/staining/Lung-HE/CAG+L╖╬_1.0x.jpg]

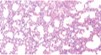

Supplement: Supplementary file 1 [file DataSheet1.ZIP › Original data/staining/Lung-HE/CAG+L╖╬_20.0x.jpg]

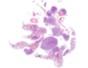

Supplement: Supplementary file 1 [file DataSheet1.ZIP › Original data/staining/Lung-HE/CAG╖╬_1.0x.jpg]

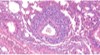

Supplement: Supplementary file 1 [file DataSheet1.ZIP › Original data/staining/Lung-HE/CAG╖╬_20.0x.jpg]

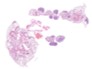

Supplement: Supplementary file 1 [file DataSheet1.ZIP › Original data/staining/Lung-HE/CA╖╬_1.0x.jpg]

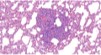

Supplement: Supplementary file 1 [file DataSheet1.ZIP › Original data/staining/Lung-HE/CA╖╬_20.0x.jpg]

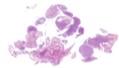

Supplement: Supplementary file 1 [file DataSheet1.ZIP › Original data/staining/Lung-HE/PBS+L ╖╬_1.0x.jpg]

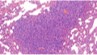

Supplement: Supplementary file 1 [file DataSheet1.ZIP › Original data/staining/Lung-HE/PBS+L ╖╬_20.0x.jpg]

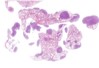

Supplement: Supplementary file 1 [file DataSheet1.ZIP › Original data/staining/Lung-HE/PBS╖╬_1.0x.jpg]

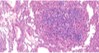

Supplement: Supplementary file 1 [file DataSheet1.ZIP › Original data/staining/Lung-HE/PBS╖╬_20.0x.jpg]

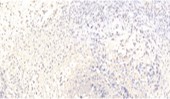

Supplement: Supplementary file 1 [file DataSheet1.ZIP › Original data/staining/TUNEL/CA+L.jpg]

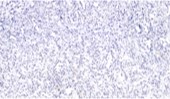

Supplement: Supplementary file 1 [file DataSheet1.ZIP › Original data/staining/TUNEL/CA.jpg]

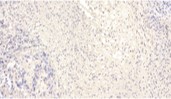

Supplement: Supplementary file 1 [file DataSheet1.ZIP › Original data/staining/TUNEL/CAG+L.jpg]

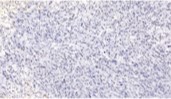

Supplement: Supplementary file 1 [file DataSheet1.ZIP › Original data/staining/TUNEL/CAG.jpg]

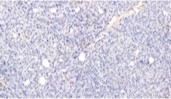

Supplement: Supplementary file 1 [file DataSheet1.ZIP › Original data/staining/TUNEL/PBS+L.jpg]

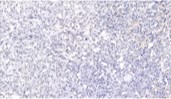

Supplement: Supplementary file 1 [file DataSheet1.ZIP › Original data/staining/TUNEL/PBS.jpg]

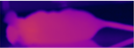

Supplement: Supplementary file 1 [file DataSheet1.ZIP › Original data/Tumor-PTT/╜╪═╝/CA1.png]

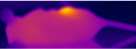

Supplement: Supplementary file 1 [file DataSheet1.ZIP › Original data/Tumor-PTT/╜╪═╝/CA2.png]

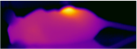

Supplement: Supplementary file 1 [file DataSheet1.ZIP › Original data/Tumor-PTT/╜╪═╝/CA3.png]

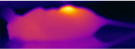

Supplement: Supplementary file 1 [file DataSheet1.ZIP › Original data/Tumor-PTT/╜╪═╝/CA4.png]

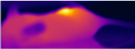

Supplement: Supplementary file 1 [file DataSheet1.ZIP › Original data/Tumor-PTT/╜╪═╝/CA5.png]

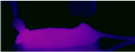

Supplement: Supplementary file 1 [file DataSheet1.ZIP › Original data/Tumor-PTT/╜╪═╝/CAG1.png]

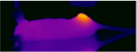

Supplement: Supplementary file 1 [file DataSheet1.ZIP › Original data/Tumor-PTT/╜╪═╝/CAG2.png]

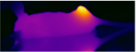

Supplement: Supplementary file 1 [file DataSheet1.ZIP › Original data/Tumor-PTT/╜╪═╝/CAG3.png]

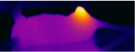

Supplement: Supplementary file 1 [file DataSheet1.ZIP › Original data/Tumor-PTT/╜╪═╝/CAG4.png]

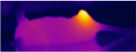

Supplement: Supplementary file 1 [file DataSheet1.ZIP › Original data/Tumor-PTT/╜╪═╝/CAG5.png]

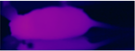

Supplement: Supplementary file 1 [file DataSheet1.ZIP › Original data/Tumor-PTT/╜╪═╝/PBS1.png]

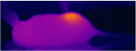

Supplement: Supplementary file 1 [file DataSheet1.ZIP › Original data/Tumor-PTT/╜╪═╝/PBS2.png]

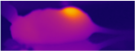

Supplement: Supplementary file 1 [file DataSheet1.ZIP › Original data/Tumor-PTT/╜╪═╝/PBS3.png]

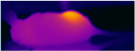

Supplement: Supplementary file 1 [file DataSheet1.ZIP › Original data/Tumor-PTT/╜╪═╝/PBS4.png]

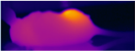

Supplement: Supplementary file 1 [file DataSheet1.ZIP › Original data/Tumor-PTT/╜╪═╝/PBS5.png]
