# Supplementary figures and images for: Enzyme-Loaded pH-Sensitive Photothermal Hydrogels for Mild-temperature-mediated Combinational Cancer Therapy (part 1 of 2)
Source: Front Chem. 2021 Jul 29;9:736468. doi: 10.3389/fchem.2021.736468 (PMC8358069; doi:10.3389/fchem.2021.736468)

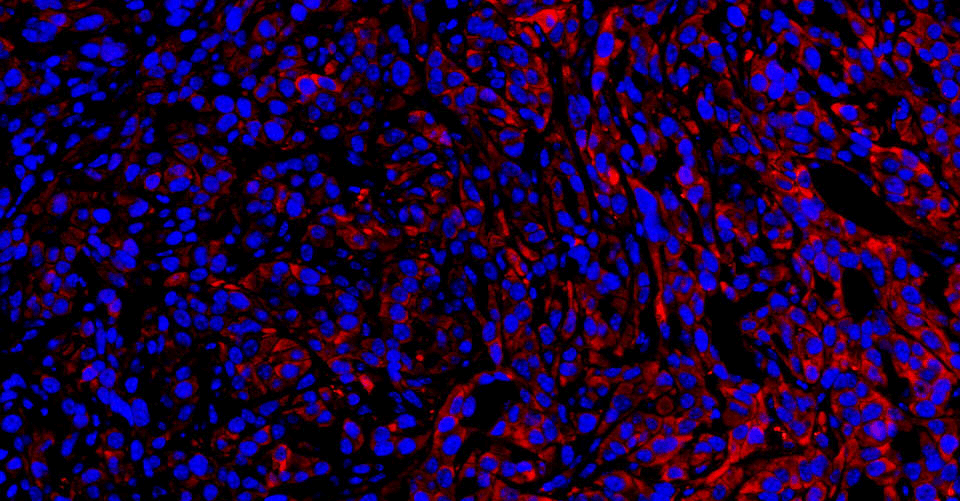

Supplement: Supplementary file 1 [file DataSheet1.ZIP › Original data/HSP90 expression/images/CA+L_01.gif]

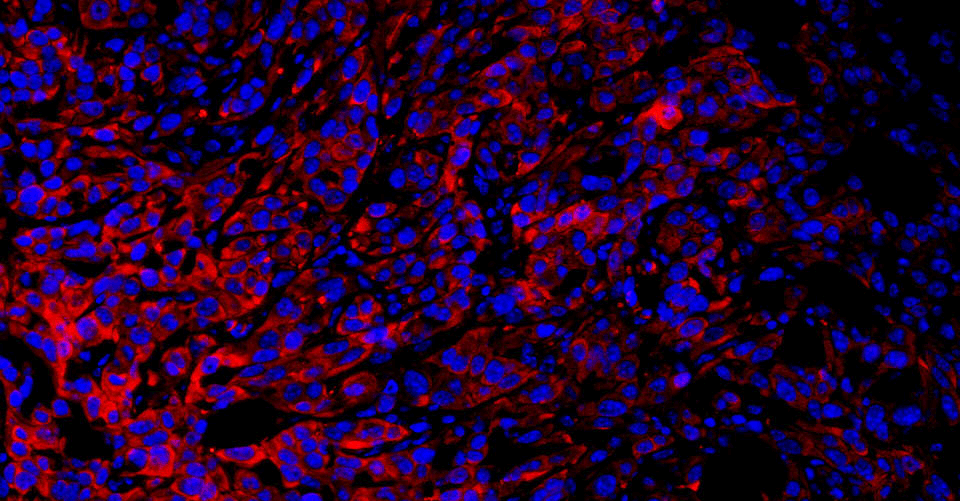

Supplement: Supplementary file 1 [file DataSheet1.ZIP › Original data/HSP90 expression/images/CA+L_02.gif]

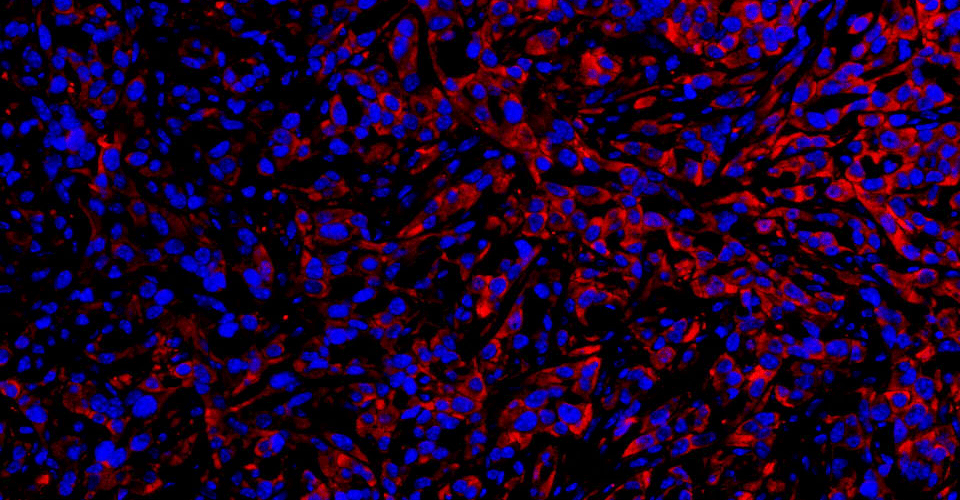

Supplement: Supplementary file 1 [file DataSheet1.ZIP › Original data/HSP90 expression/images/CA+L_03.gif]

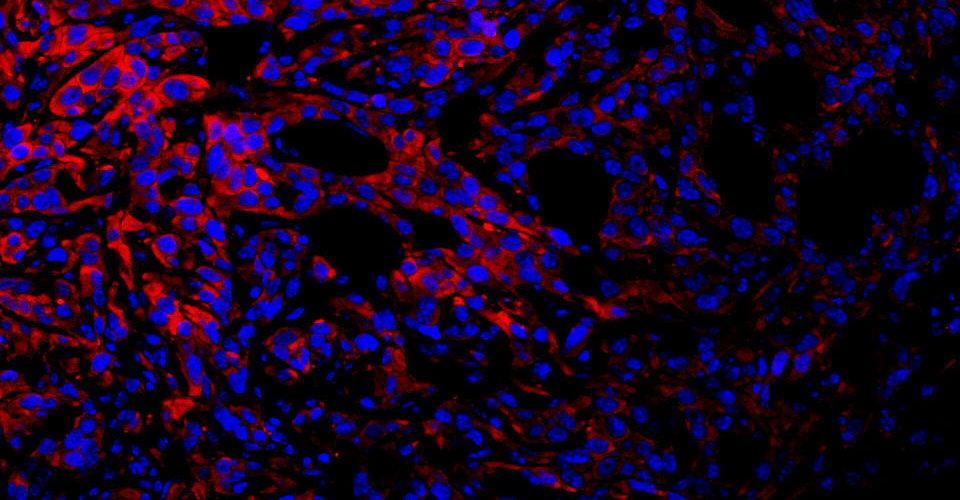

Supplement: Supplementary file 1 [file DataSheet1.ZIP › Original data/HSP90 expression/images/CA+L_04.gif]

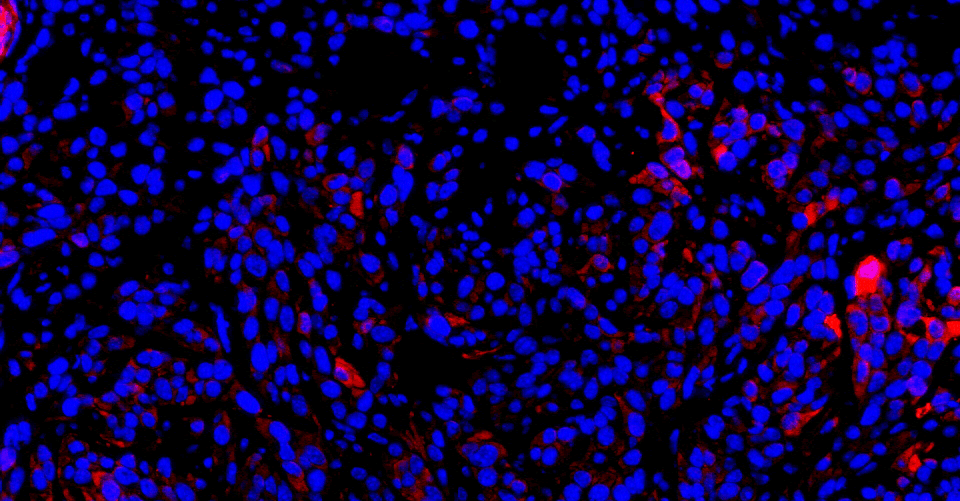

Supplement: Supplementary file 1 [file DataSheet1.ZIP › Original data/HSP90 expression/images/CAG+L_01.gif]

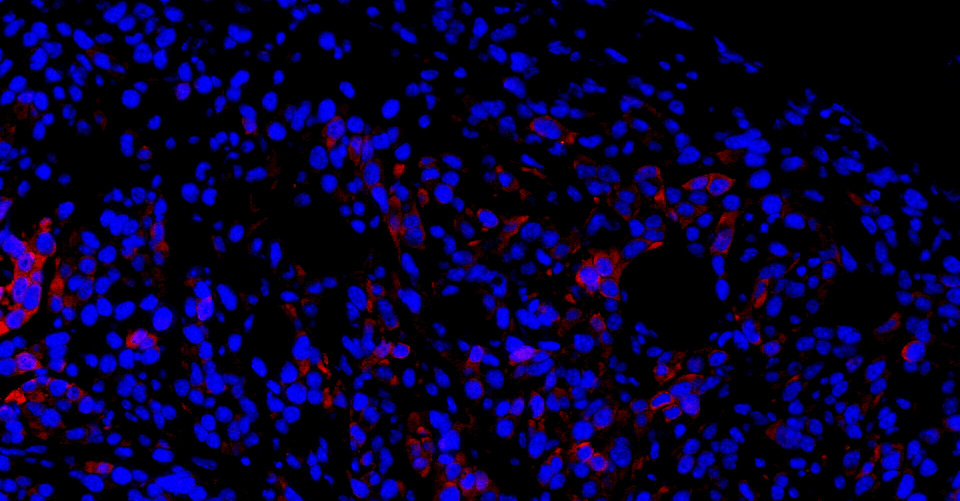

Supplement: Supplementary file 1 [file DataSheet1.ZIP › Original data/HSP90 expression/images/CAG+L_02.gif]

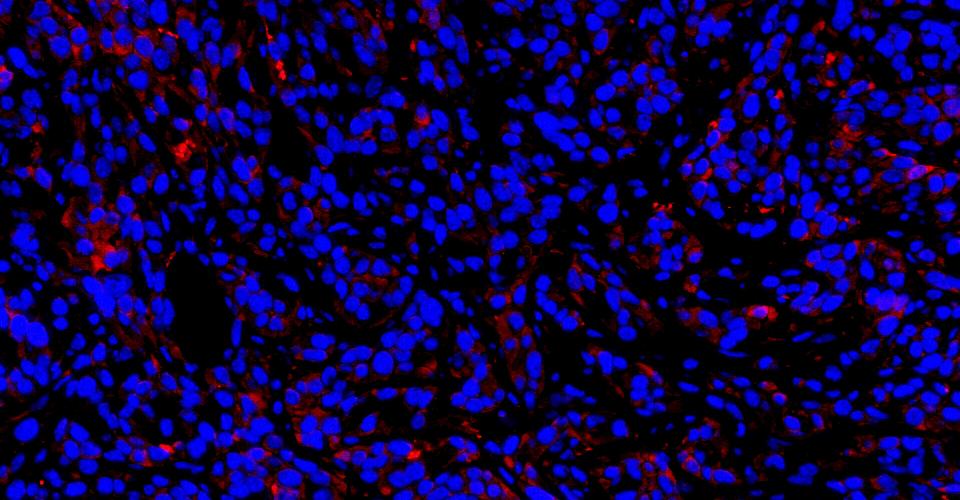

Supplement: Supplementary file 1 [file DataSheet1.ZIP › Original data/HSP90 expression/images/CAG+L_03.gif]

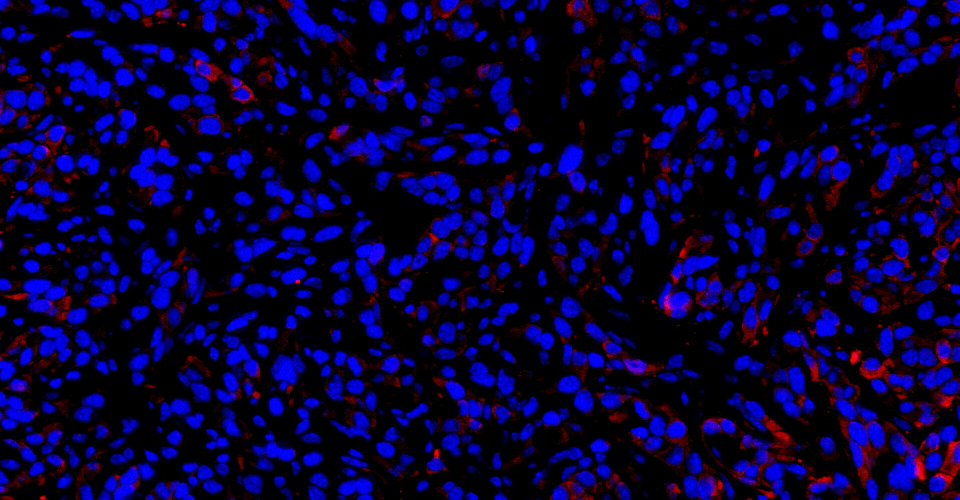

Supplement: Supplementary file 1 [file DataSheet1.ZIP › Original data/HSP90 expression/images/CAG+L_04.gif]

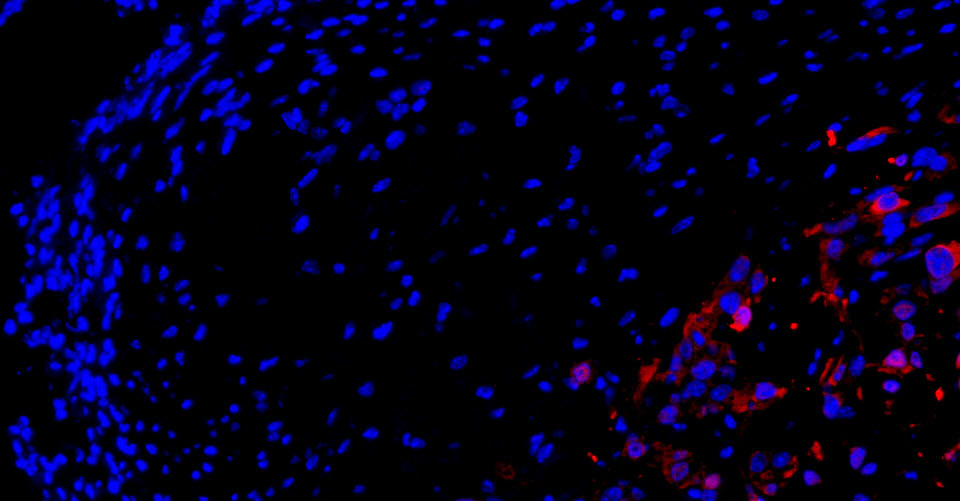

Supplement: Supplementary file 1 [file DataSheet1.ZIP › Original data/HSP90 expression/images/CAG_01.gif]

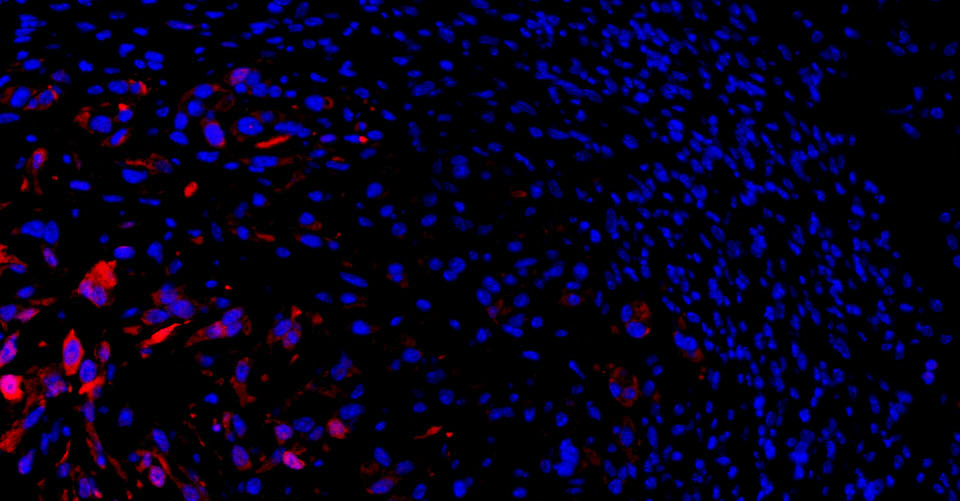

Supplement: Supplementary file 1 [file DataSheet1.ZIP › Original data/HSP90 expression/images/CAG_02.gif]

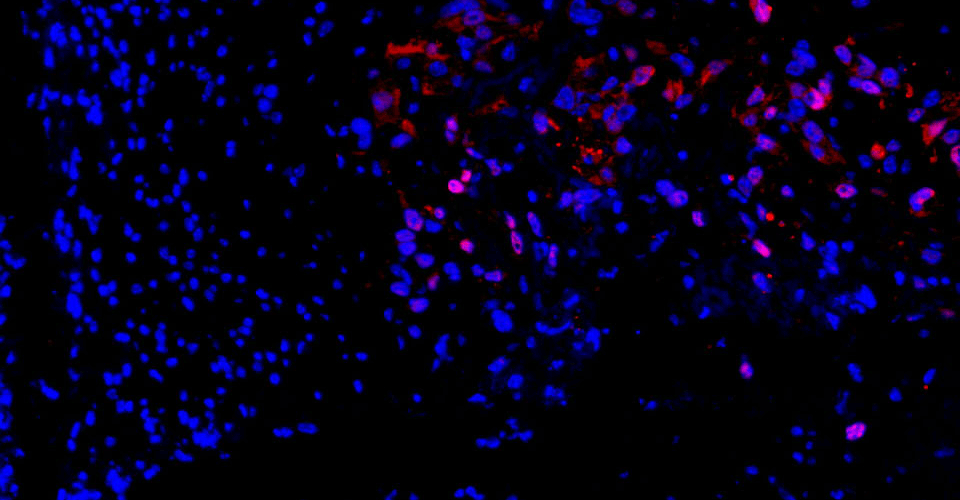

Supplement: Supplementary file 1 [file DataSheet1.ZIP › Original data/HSP90 expression/images/CAG_03.gif]

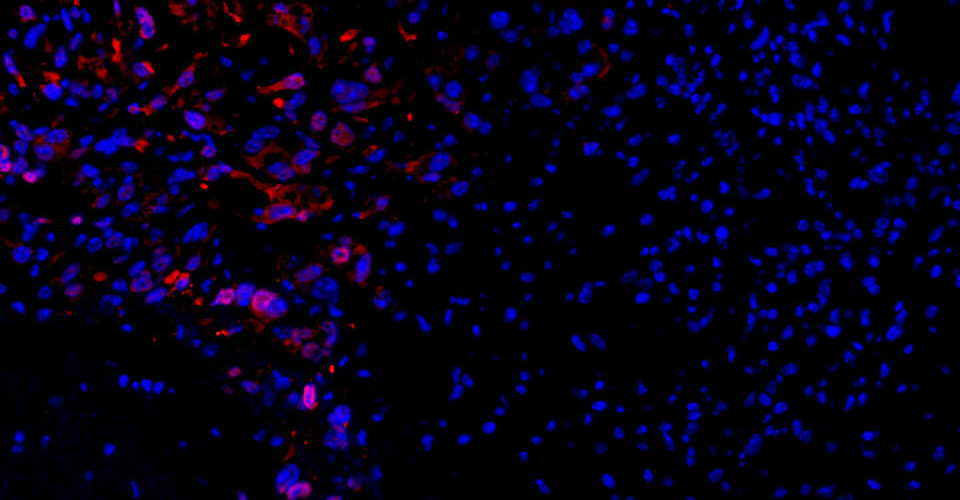

Supplement: Supplementary file 1 [file DataSheet1.ZIP › Original data/HSP90 expression/images/CAG_04.gif]

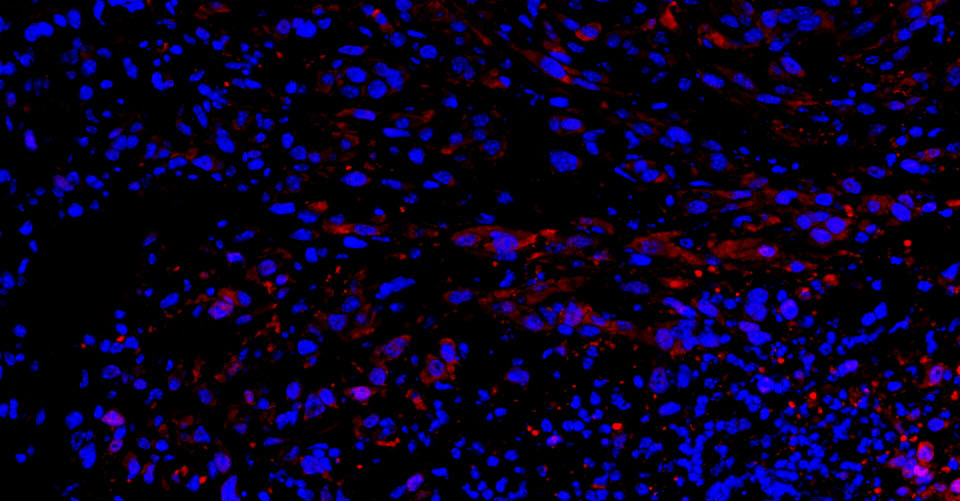

Supplement: Supplementary file 1 [file DataSheet1.ZIP › Original data/HSP90 expression/images/CA_01.gif]

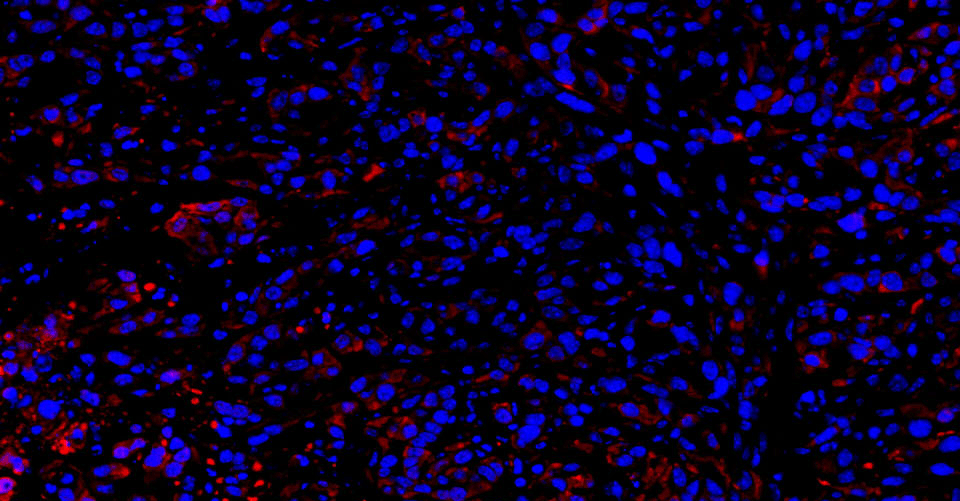

Supplement: Supplementary file 1 [file DataSheet1.ZIP › Original data/HSP90 expression/images/CA_02.gif]

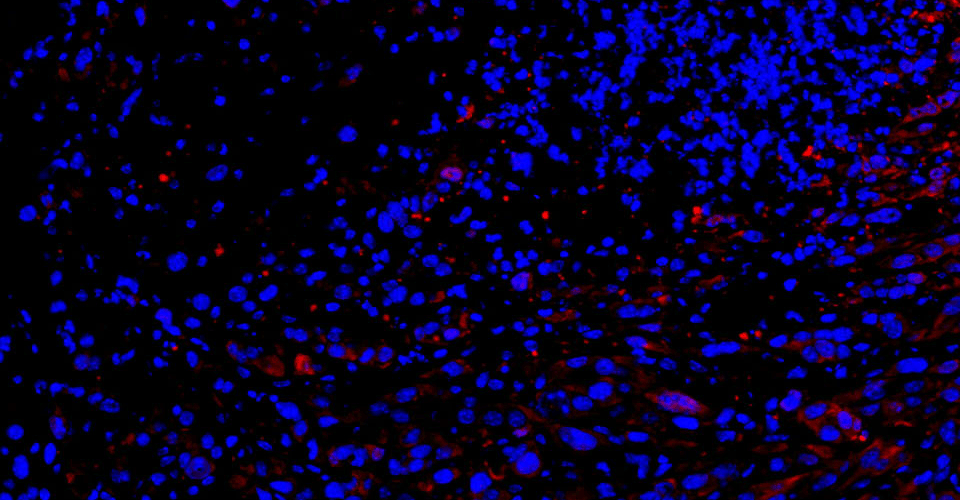

Supplement: Supplementary file 1 [file DataSheet1.ZIP › Original data/HSP90 expression/images/CA_03.gif]

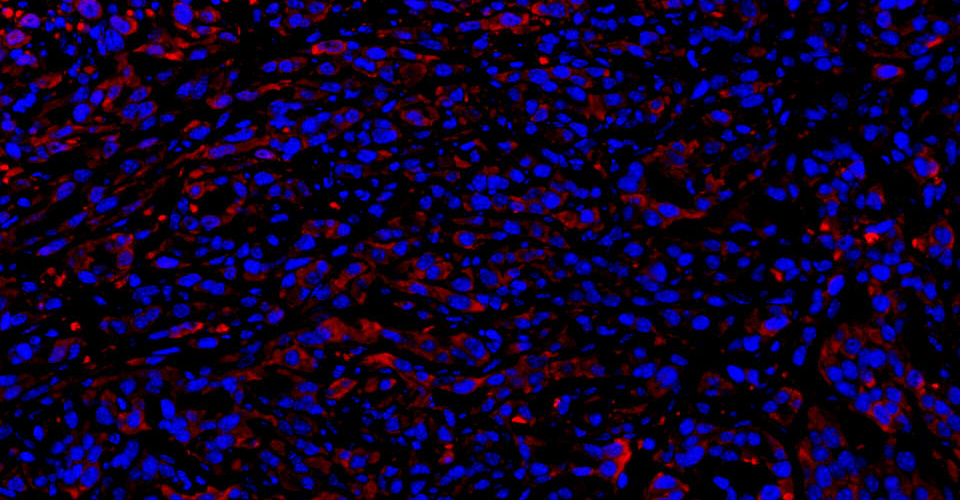

Supplement: Supplementary file 1 [file DataSheet1.ZIP › Original data/HSP90 expression/images/CA_04.gif]

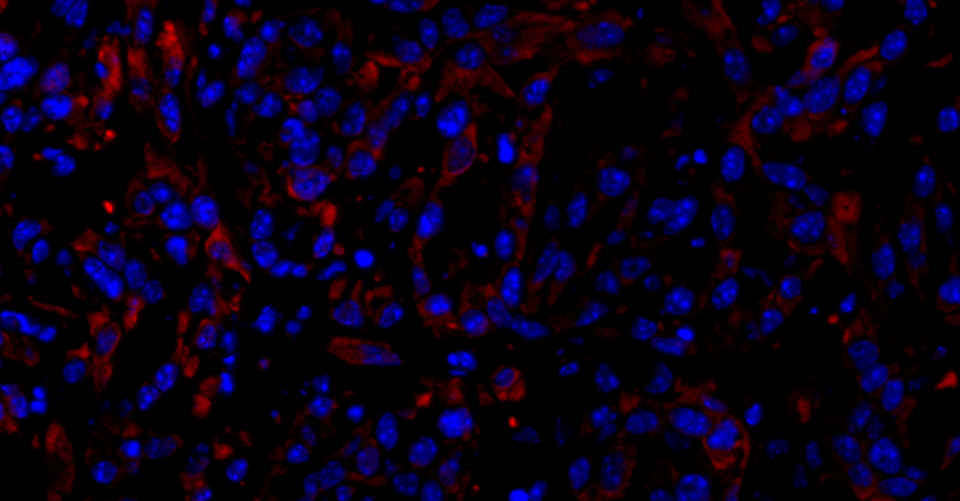

Supplement: Supplementary file 1 [file DataSheet1.ZIP › Original data/HSP90 expression/images/pbs+l-1 (1).gif]

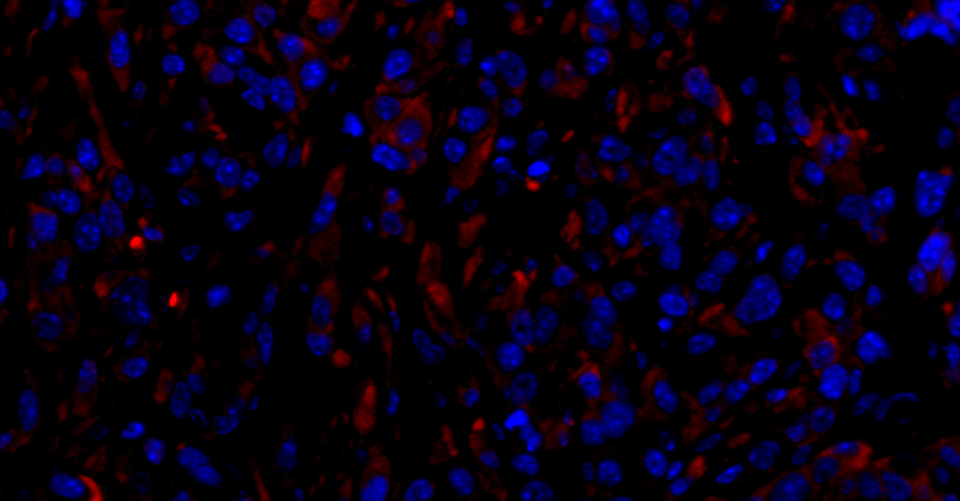

Supplement: Supplementary file 1 [file DataSheet1.ZIP › Original data/HSP90 expression/images/pbs+l-1 (2).gif]

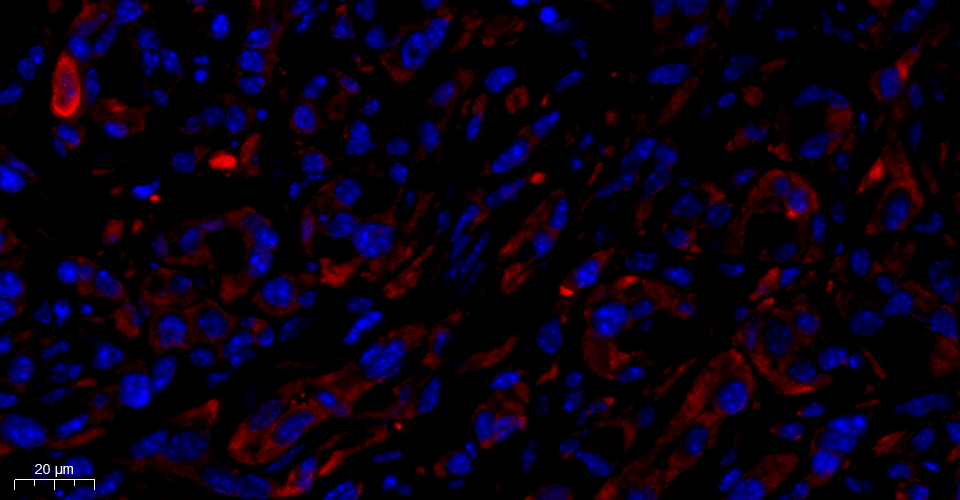

Supplement: Supplementary file 1 [file DataSheet1.ZIP › Original data/HSP90 expression/images/pbs+l-1 (3).gif]

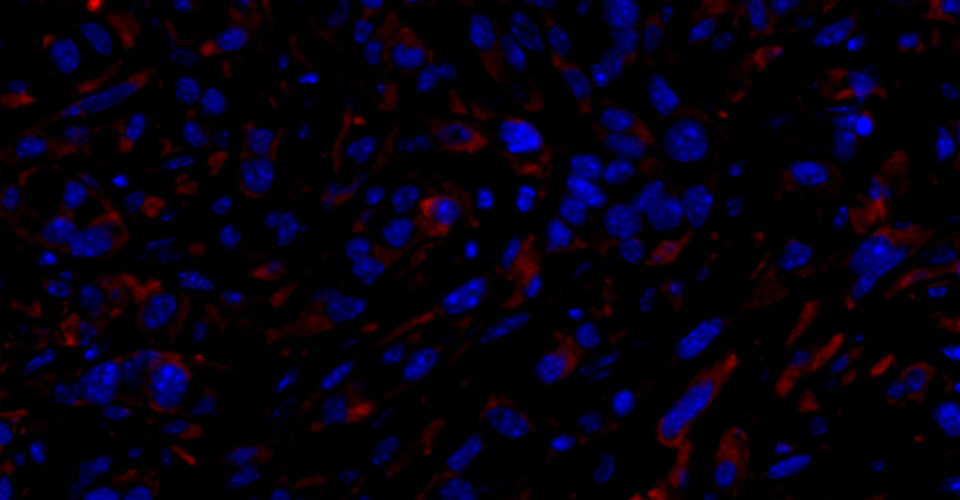

Supplement: Supplementary file 1 [file DataSheet1.ZIP › Original data/HSP90 expression/images/pbs+l-1 (4).gif]

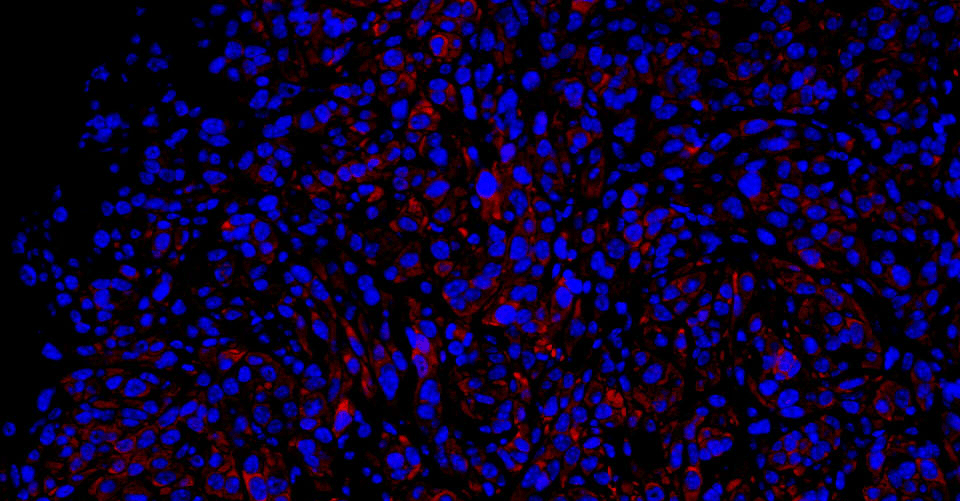

Supplement: Supplementary file 1 [file DataSheet1.ZIP › Original data/HSP90 expression/images/PBS_01.gif]

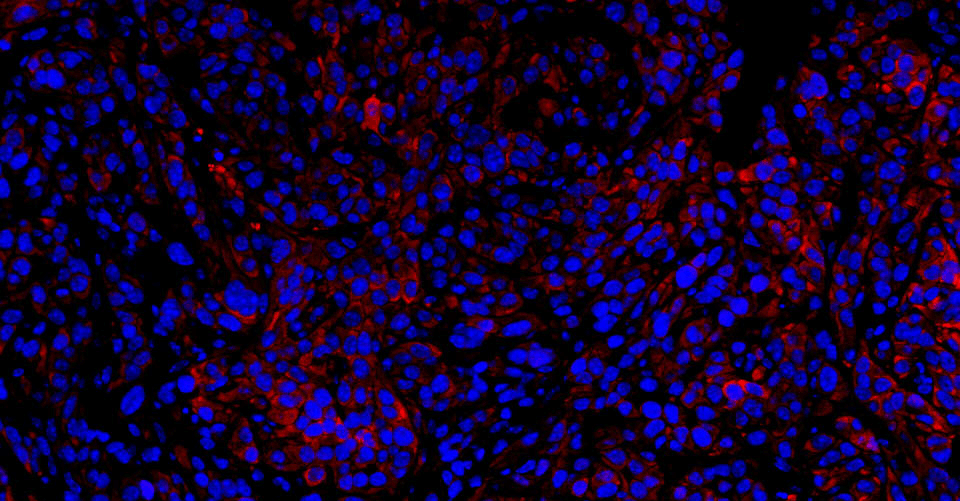

Supplement: Supplementary file 1 [file DataSheet1.ZIP › Original data/HSP90 expression/images/PBS_02.gif]

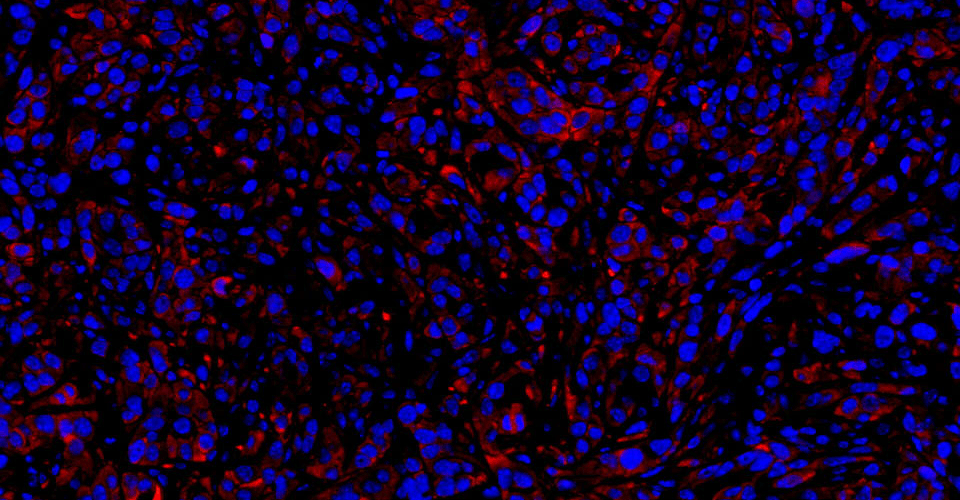

Supplement: Supplementary file 1 [file DataSheet1.ZIP › Original data/HSP90 expression/images/PBS_03.gif]

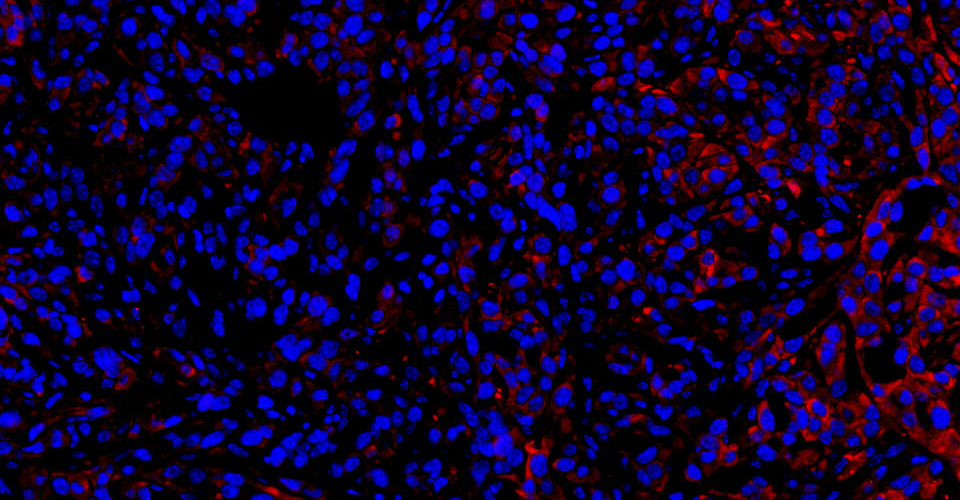

Supplement: Supplementary file 1 [file DataSheet1.ZIP › Original data/HSP90 expression/images/PBS_04.gif]

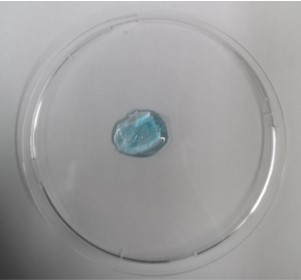

Supplement: Supplementary file 1 [file DataSheet1.ZIP › Original data/Hydrogel formation/CA.jpg]

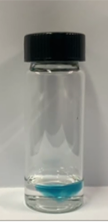

Supplement: Supplementary file 1 [file DataSheet1.ZIP › Original data/Hydrogel formation/CA.png]

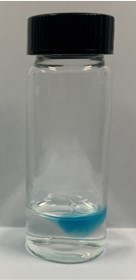

Supplement: Supplementary file 1 [file DataSheet1.ZIP › Original data/Hydrogel formation/CAG-2.jpg]

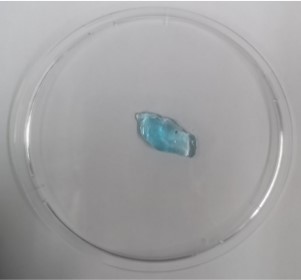

Supplement: Supplementary file 1 [file DataSheet1.ZIP › Original data/Hydrogel formation/CAG.jpg]

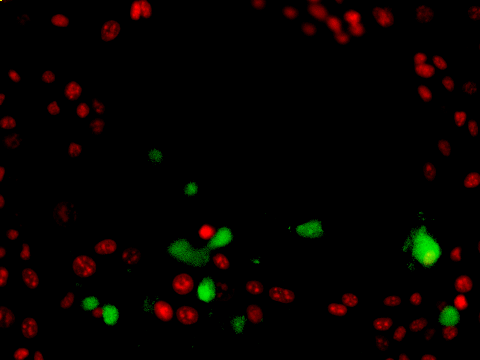

Supplement: Supplementary file 1 [file DataSheet1.ZIP › Original data/live and dead staining/images/CA+LASER_01.gif]

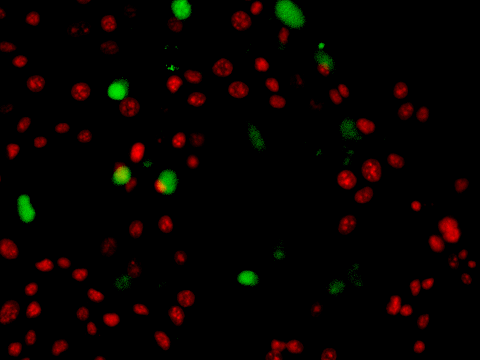

Supplement: Supplementary file 1 [file DataSheet1.ZIP › Original data/live and dead staining/images/CA+LASER_02.gif]

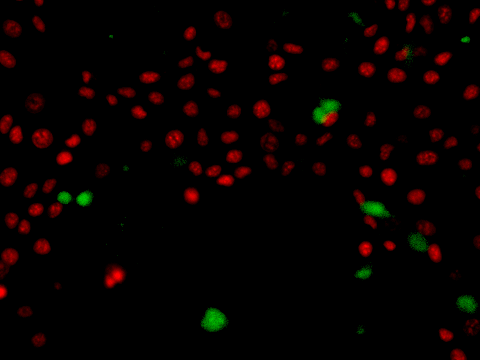

Supplement: Supplementary file 1 [file DataSheet1.ZIP › Original data/live and dead staining/images/CA+LASER_03.gif]

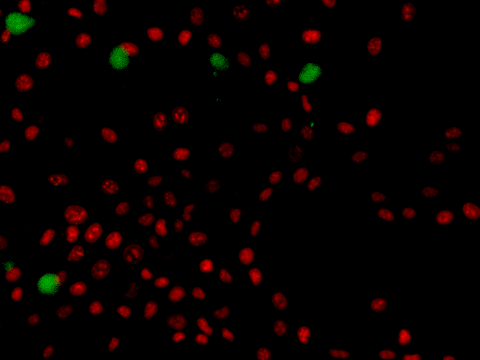

Supplement: Supplementary file 1 [file DataSheet1.ZIP › Original data/live and dead staining/images/CA+LASER_04.gif]

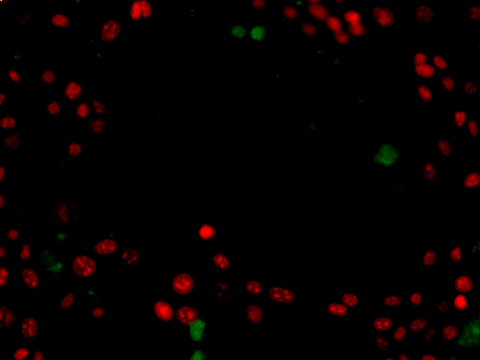

Supplement: Supplementary file 1 [file DataSheet1.ZIP › Original data/live and dead staining/images/CAG+L_01.gif]

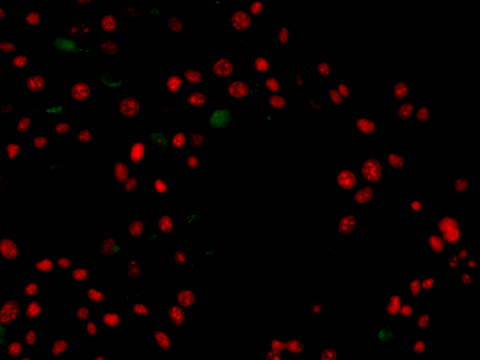

Supplement: Supplementary file 1 [file DataSheet1.ZIP › Original data/live and dead staining/images/CAG+L_02.gif]

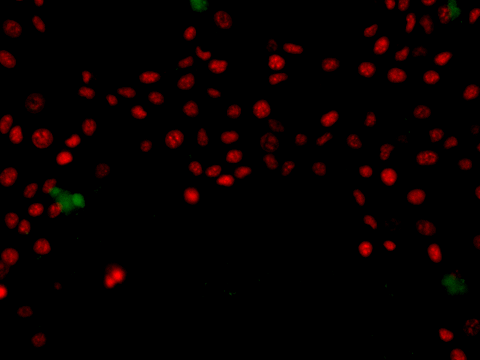

Supplement: Supplementary file 1 [file DataSheet1.ZIP › Original data/live and dead staining/images/CAG+L_03.gif]

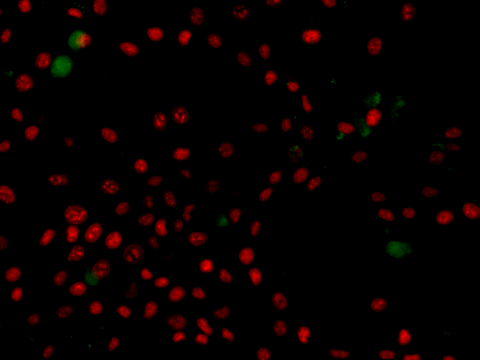

Supplement: Supplementary file 1 [file DataSheet1.ZIP › Original data/live and dead staining/images/CAG+L_04.gif]

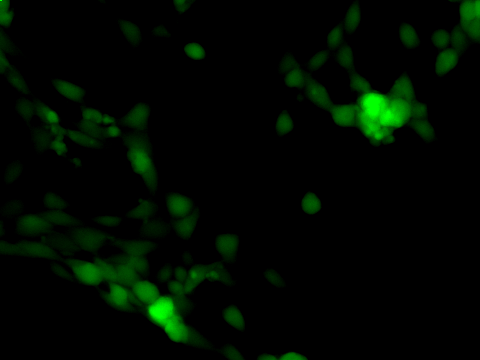

Supplement: Supplementary file 1 [file DataSheet1.ZIP › Original data/live and dead staining/images/CAG_01.gif]

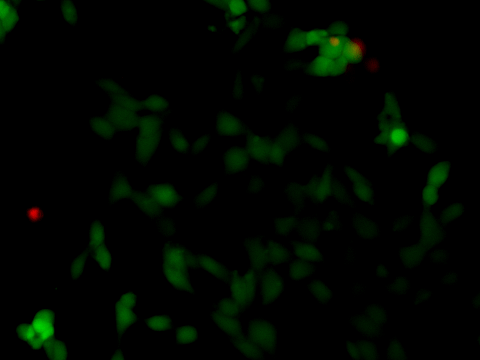

Supplement: Supplementary file 1 [file DataSheet1.ZIP › Original data/live and dead staining/images/CAG_02.gif]

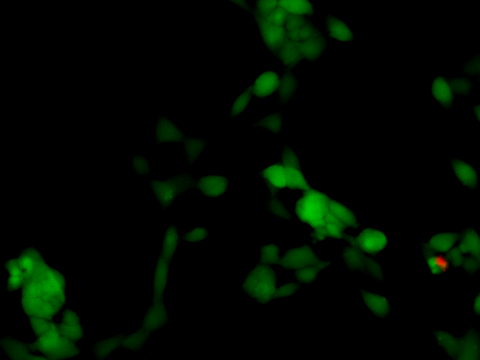

Supplement: Supplementary file 1 [file DataSheet1.ZIP › Original data/live and dead staining/images/CAG_03.gif]

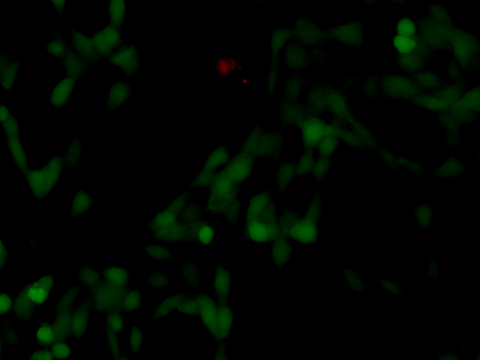

Supplement: Supplementary file 1 [file DataSheet1.ZIP › Original data/live and dead staining/images/CAG_04.gif]

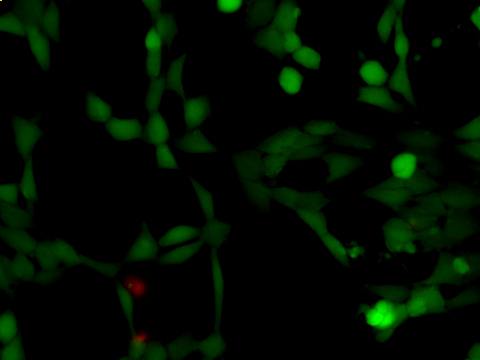

Supplement: Supplementary file 1 [file DataSheet1.ZIP › Original data/live and dead staining/images/CA_01.gif]

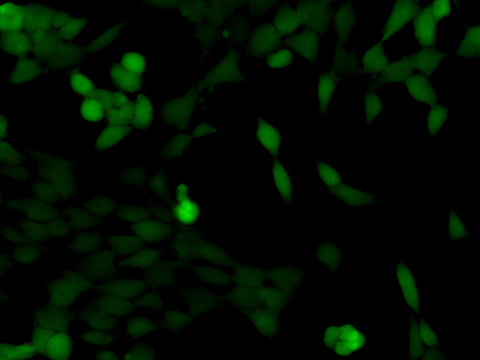

Supplement: Supplementary file 1 [file DataSheet1.ZIP › Original data/live and dead staining/images/CA_02.gif]

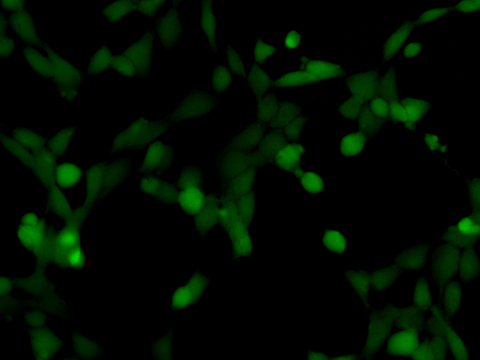

Supplement: Supplementary file 1 [file DataSheet1.ZIP › Original data/live and dead staining/images/CA_03.gif]

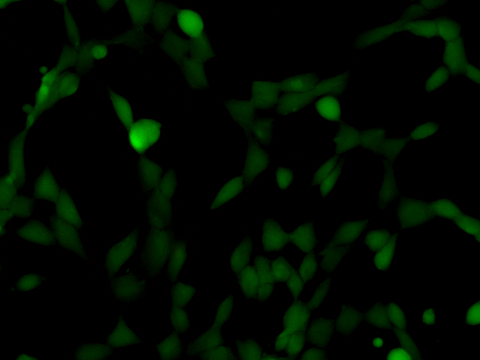

Supplement: Supplementary file 1 [file DataSheet1.ZIP › Original data/live and dead staining/images/CA_04.gif]

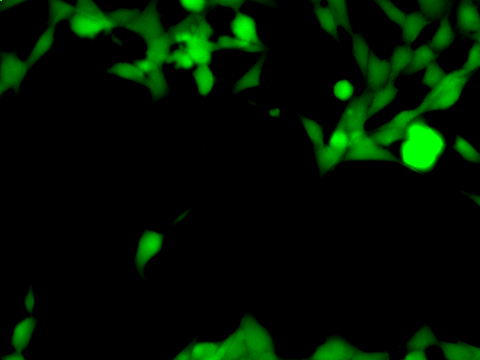

Supplement: Supplementary file 1 [file DataSheet1.ZIP › Original data/live and dead staining/images/PBS+L_01.gif]

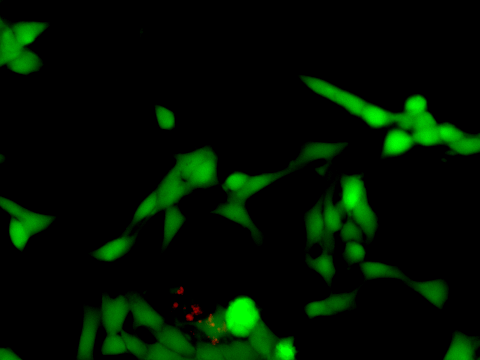

Supplement: Supplementary file 1 [file DataSheet1.ZIP › Original data/live and dead staining/images/PBS+L_02.gif]

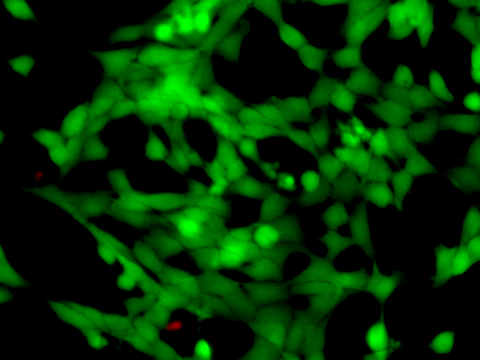

Supplement: Supplementary file 1 [file DataSheet1.ZIP › Original data/live and dead staining/images/PBS+L_03.gif]

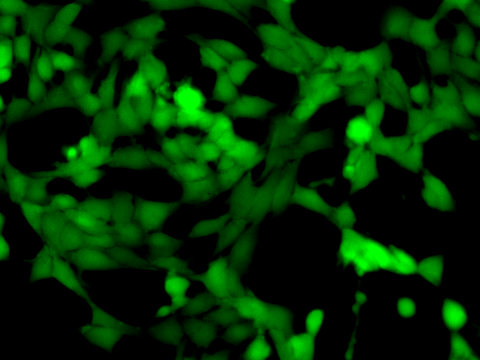

Supplement: Supplementary file 1 [file DataSheet1.ZIP › Original data/live and dead staining/images/PBS+L_04.gif]

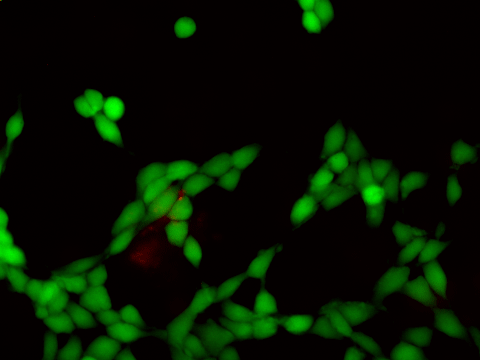

Supplement: Supplementary file 1 [file DataSheet1.ZIP › Original data/live and dead staining/images/PBS_01.gif]

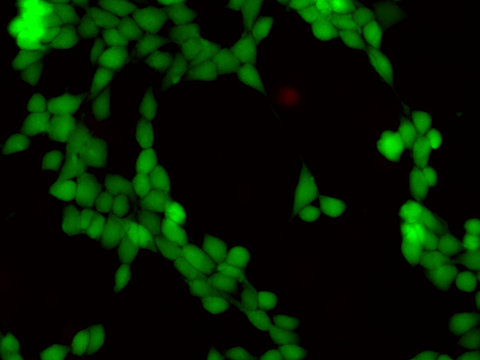

Supplement: Supplementary file 1 [file DataSheet1.ZIP › Original data/live and dead staining/images/PBS_02.gif]

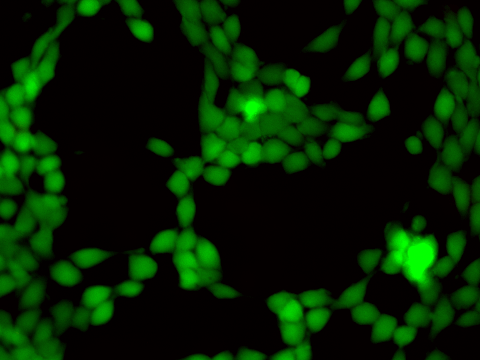

Supplement: Supplementary file 1 [file DataSheet1.ZIP › Original data/live and dead staining/images/PBS_03.gif]

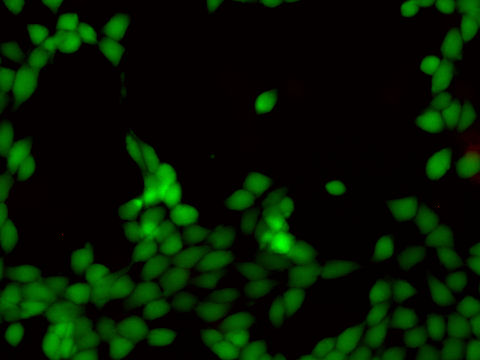

Supplement: Supplementary file 1 [file DataSheet1.ZIP › Original data/live and dead staining/images/PBS_04.gif]

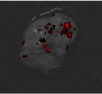

Supplement: Supplementary file 1 [file DataSheet1.ZIP › Original data/Lung metastasis/│╔╧±/CA+l-╖╬.jpg.png]

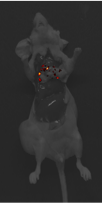

Supplement: Supplementary file 1 [file DataSheet1.ZIP › Original data/Lung metastasis/│╔╧±/CA+L.png]

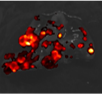

Supplement: Supplementary file 1 [file DataSheet1.ZIP › Original data/Lung metastasis/│╔╧±/CA-╖╬.jpg.png]

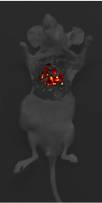

Supplement: Supplementary file 1 [file DataSheet1.ZIP › Original data/Lung metastasis/│╔╧±/CA.png]

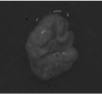

Supplement: Supplementary file 1 [file DataSheet1.ZIP › Original data/Lung metastasis/│╔╧±/CAG+L-╖╬.png]

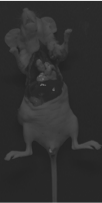

Supplement: Supplementary file 1 [file DataSheet1.ZIP › Original data/Lung metastasis/│╔╧±/CAG+L.png]

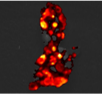

Supplement: Supplementary file 1 [file DataSheet1.ZIP › Original data/Lung metastasis/│╔╧±/CAG-╖╬.jpg.png]

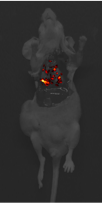

Supplement: Supplementary file 1 [file DataSheet1.ZIP › Original data/Lung metastasis/│╔╧±/CAG.png]

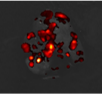

Supplement: Supplementary file 1 [file DataSheet1.ZIP › Original data/Lung metastasis/│╔╧±/PBS+L-╖╬.jpg.png]

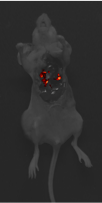

Supplement: Supplementary file 1 [file DataSheet1.ZIP › Original data/Lung metastasis/│╔╧±/PBS+L.png]

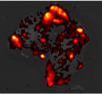

Supplement: Supplementary file 1 [file DataSheet1.ZIP › Original data/Lung metastasis/│╔╧±/PBS-╖╬.png]

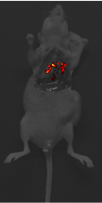

Supplement: Supplementary file 1 [file DataSheet1.ZIP › Original data/Lung metastasis/│╔╧±/PBS.png]

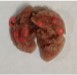

Supplement: Supplementary file 1 [file DataSheet1.ZIP › Original data/Lung metastasis/└δ╠σ╖╬/CA+L.jpg]

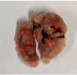

Supplement: Supplementary file 1 [file DataSheet1.ZIP › Original data/Lung metastasis/└δ╠σ╖╬/CA.jpg]

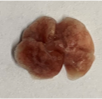

Supplement: Supplementary file 1 [file DataSheet1.ZIP › Original data/Lung metastasis/└δ╠σ╖╬/cag+l.png]

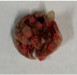

Supplement: Supplementary file 1 [file DataSheet1.ZIP › Original data/Lung metastasis/└δ╠σ╖╬/CAG.jpg]

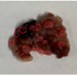

Supplement: Supplementary file 1 [file DataSheet1.ZIP › Original data/Lung metastasis/└δ╠σ╖╬/PBS+L.jpg]

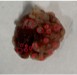

Supplement: Supplementary file 1 [file DataSheet1.ZIP › Original data/Lung metastasis/└δ╠σ╖╬/PBS.jpg]

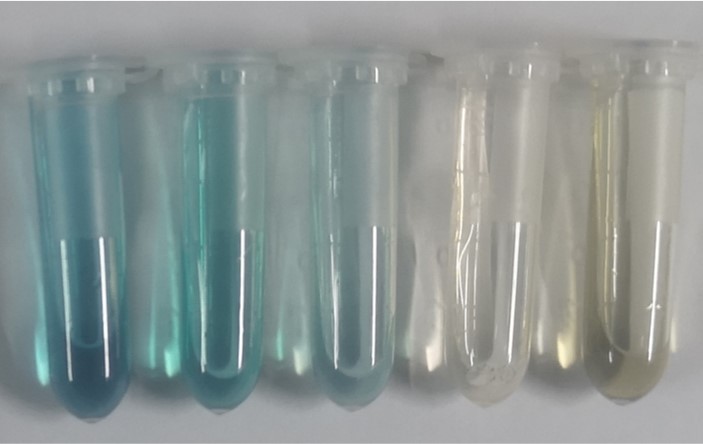

Supplement: Supplementary file 1 [file DataSheet1.ZIP › Original data/ph-UV/═╝╞1⁄4.jpg]

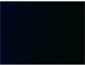

Supplement: Supplementary file 1 [file DataSheet1.ZIP › Original data/photothermal property/╒╒╞1⁄4/CA0.png]

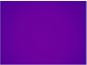

Supplement: Supplementary file 1 [file DataSheet1.ZIP › Original data/photothermal property/╒╒╞1⁄4/CA1.png]

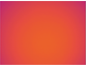

Supplement: Supplementary file 1 [file DataSheet1.ZIP › Original data/photothermal property/╒╒╞1⁄4/CA2.png]

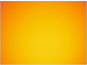

Supplement: Supplementary file 1 [file DataSheet1.ZIP › Original data/photothermal property/╒╒╞1⁄4/CA3.png]

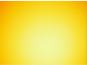

Supplement: Supplementary file 1 [file DataSheet1.ZIP › Original data/photothermal property/╒╒╞1⁄4/CA4.png]

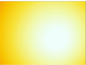

Supplement: Supplementary file 1 [file DataSheet1.ZIP › Original data/photothermal property/╒╒╞1⁄4/CA5.png]

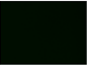

Supplement: Supplementary file 1 [file DataSheet1.ZIP › Original data/photothermal property/╒╒╞1⁄4/CAG0.png]

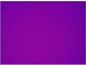

Supplement: Supplementary file 1 [file DataSheet1.ZIP › Original data/photothermal property/╒╒╞1⁄4/CAG1.png]

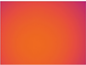

Supplement: Supplementary file 1 [file DataSheet1.ZIP › Original data/photothermal property/╒╒╞1⁄4/CAG2.png]

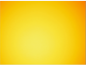

Supplement: Supplementary file 1 [file DataSheet1.ZIP › Original data/photothermal property/╒╒╞1⁄4/CAG3.png]

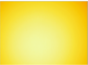

Supplement: Supplementary file 1 [file DataSheet1.ZIP › Original data/photothermal property/╒╒╞1⁄4/CAG4.png]

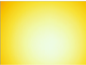

Supplement: Supplementary file 1 [file DataSheet1.ZIP › Original data/photothermal property/╒╒╞1⁄4/CAG5.png]

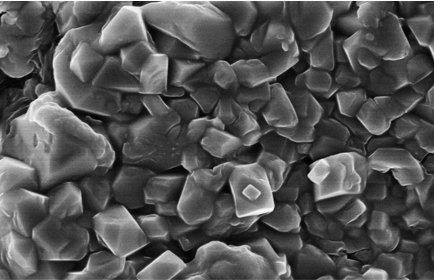

Supplement: Supplementary file 1 [file DataSheet1.ZIP › Original data/SEM/CA.tif]

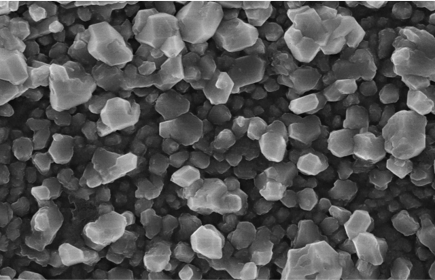

Supplement: Supplementary file 1 [file DataSheet1.ZIP › Original data/SEM/CAG.tif]

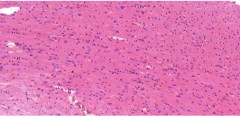

Supplement: Supplementary file 1 [file DataSheet1.ZIP › Original data/staining/HE-tissues/CA-╨─.jpg]

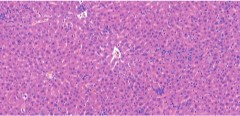

Supplement: Supplementary file 1 [file DataSheet1.ZIP › Original data/staining/HE-tissues/CA-╕╬.jpg]

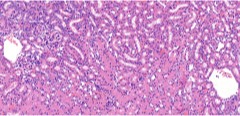

Supplement: Supplementary file 1 [file DataSheet1.ZIP › Original data/staining/HE-tissues/CA-╔÷.jpg]

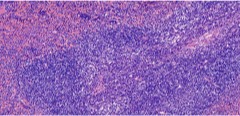

Supplement: Supplementary file 1 [file DataSheet1.ZIP › Original data/staining/HE-tissues/CA-╞ó.jpg]

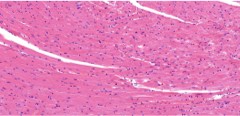

Supplement: Supplementary file 1 [file DataSheet1.ZIP › Original data/staining/HE-tissues/CAG-╨─.jpg]

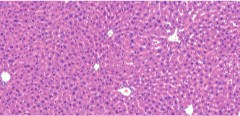

Supplement: Supplementary file 1 [file DataSheet1.ZIP › Original data/staining/HE-tissues/CAG-╕╬.jpg]

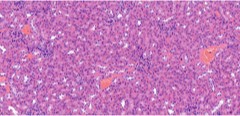

Supplement: Supplementary file 1 [file DataSheet1.ZIP › Original data/staining/HE-tissues/CAG-╔÷.jpg]

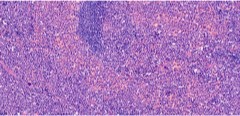

Supplement: Supplementary file 1 [file DataSheet1.ZIP › Original data/staining/HE-tissues/CAG-╞ó.jpg]

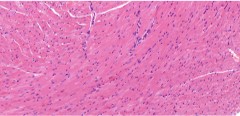

Supplement: Supplementary file 1 [file DataSheet1.ZIP › Original data/staining/HE-tissues/PBS-╨─.jpg]

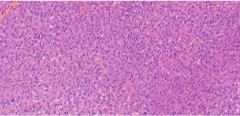

Supplement: Supplementary file 1 [file DataSheet1.ZIP › Original data/staining/HE-tissues/PBS-╕╬.jpg]

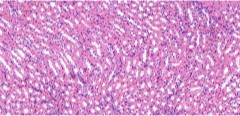

Supplement: Supplementary file 1 [file DataSheet1.ZIP › Original data/staining/HE-tissues/PBS-╔÷.jpg]

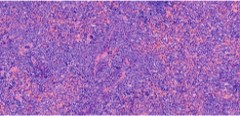

Supplement: Supplementary file 1 [file DataSheet1.ZIP › Original data/staining/HE-tissues/PBS-╞ó.jpg]

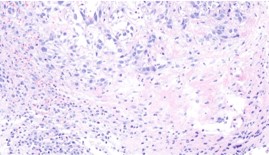

Supplement: Supplementary file 1 [file DataSheet1.ZIP › Original data/staining/HE/CA+L.jpg]

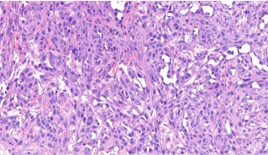

Supplement: Supplementary file 1 [file DataSheet1.ZIP › Original data/staining/HE/CA.jpg]

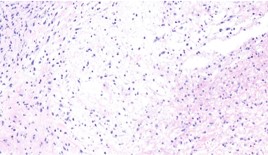

Supplement: Supplementary file 1 [file DataSheet1.ZIP › Original data/staining/HE/CAG+L.jpg]
