# Supplementary material for: Enzyme-Loaded pH-Sensitive Photothermal Hydrogels for Mild-temperature-mediated Combinational Cancer Therapy
Source: Front Chem. 2021 Jul 29;9:736468. doi: 10.3389/fchem.2021.736468 (PMC8358069; doi:10.3389/fchem.2021.736468)
Supplement: Supplementary file 2 [file DataSheet2.docx]

Supplementary Material

Enzyme-loaded pH-sensitive photothermal hydrogels for mild‐temperature-mediated combinational cancer therapy

**Jindong Xia^1#^, Xueqin Qing^2#^, Junjian Shen^3#^, Mengbin Ding^4^, Yue Wang^1^, Ningyue Yu^4^, Jingchao Li^4*^, Xiuhui Wang^5*^**

^1^Department of Radiology, Shanghai Songjiang District Central Hospital, Shanghai 201600, China

^2^Department of Pediatrics, Shanghai General Hospital, Shanghai Jiao Tong University, School of Medicine, Shanghai 200080, China

^3^Department of Radiology, The First Affiliated Hospital of Bengbu Medical College, Bengbu, Anhui 233000, China

^4^Shanghai Engineering Research Center of Nano-Biomaterials and Regenerative Medicine, College of Chemistry, Chemical Engineering and Biotechnology, Donghua University, Shanghai 201620, China

^5^Institute of Translational Medicine, Shanghai University, Shanghai 200011, China

^#^**These authors contributed equally to this work.**

*Corresponding authors: jcli@dhu.edu.cn (J. Li), wxh200801@163.com (X. Wang)

## Supplementary Figures


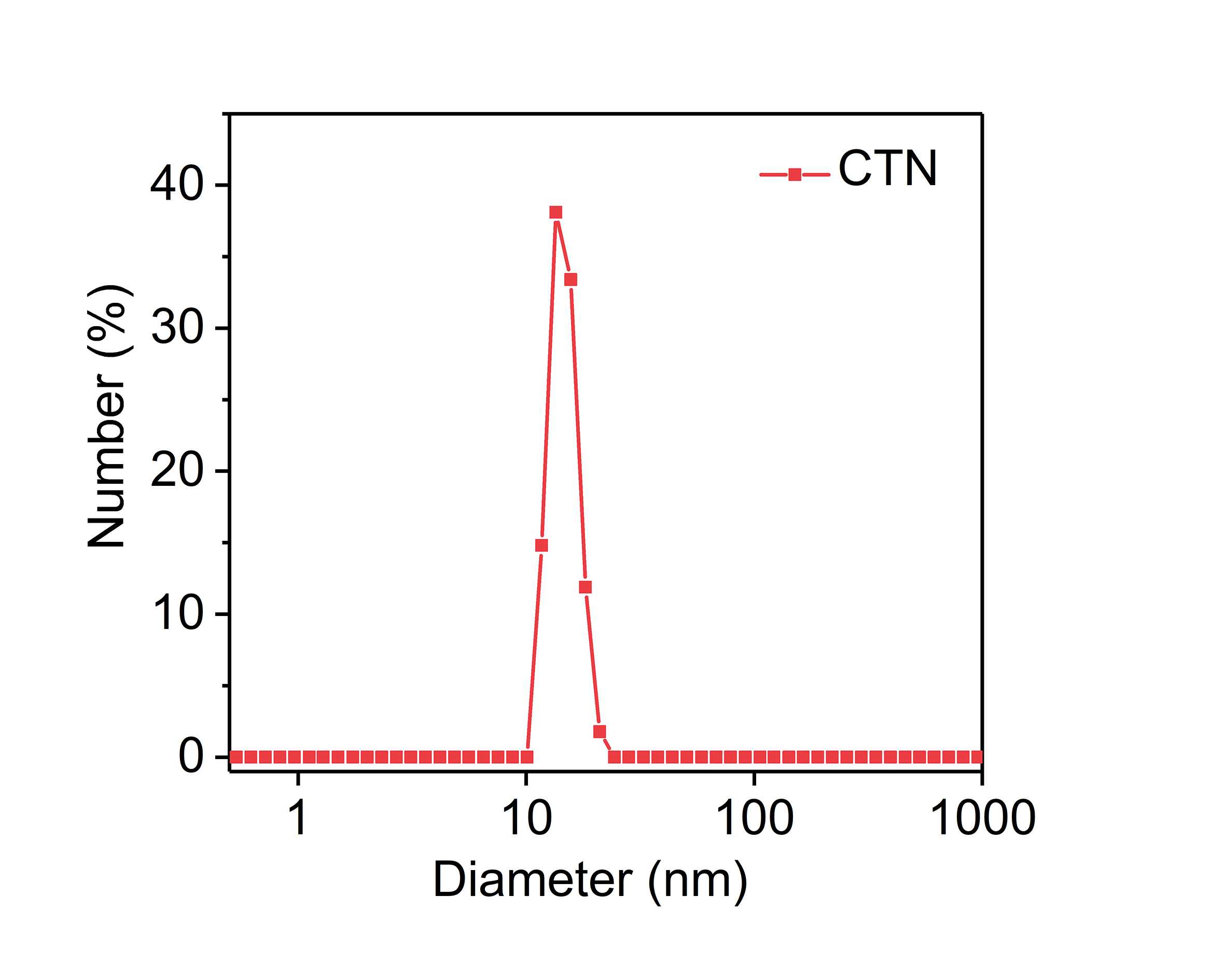


**Supplementary Figure 1.** Hydrodynamic diameter of CTNs measured by DLS.

**
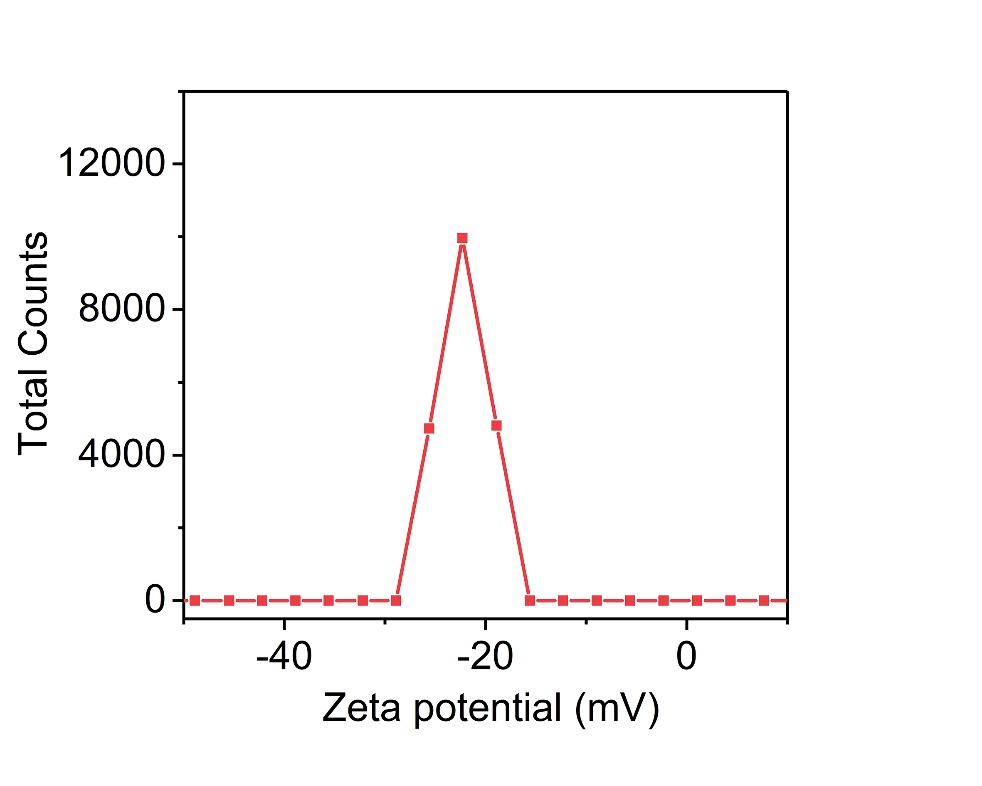
**

**Supplementary Figure 2.** Zeta potential of CTNs.


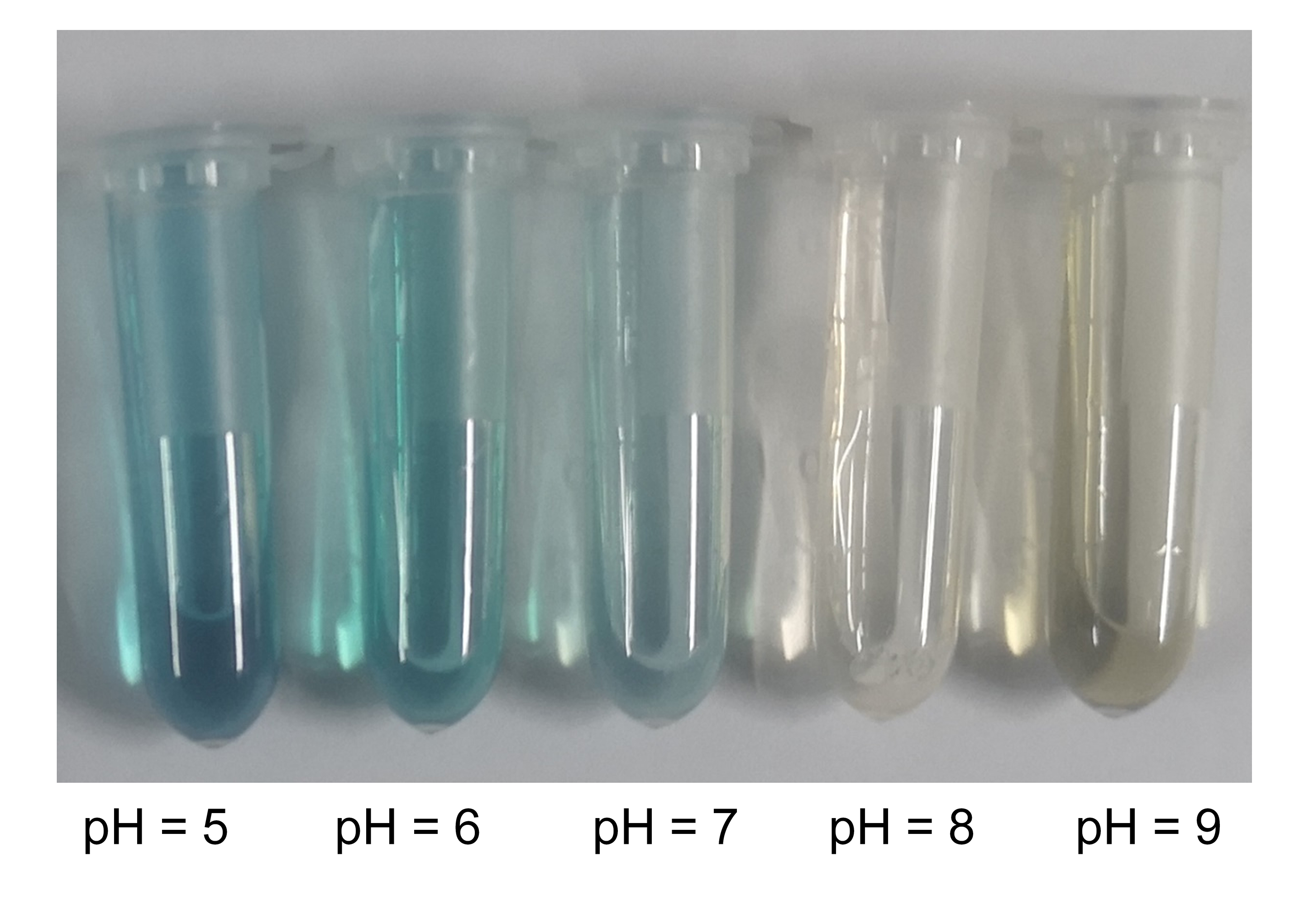


**Supplementary Figure 3.** Photographs of solutions containing CTNs at different pH conditions.


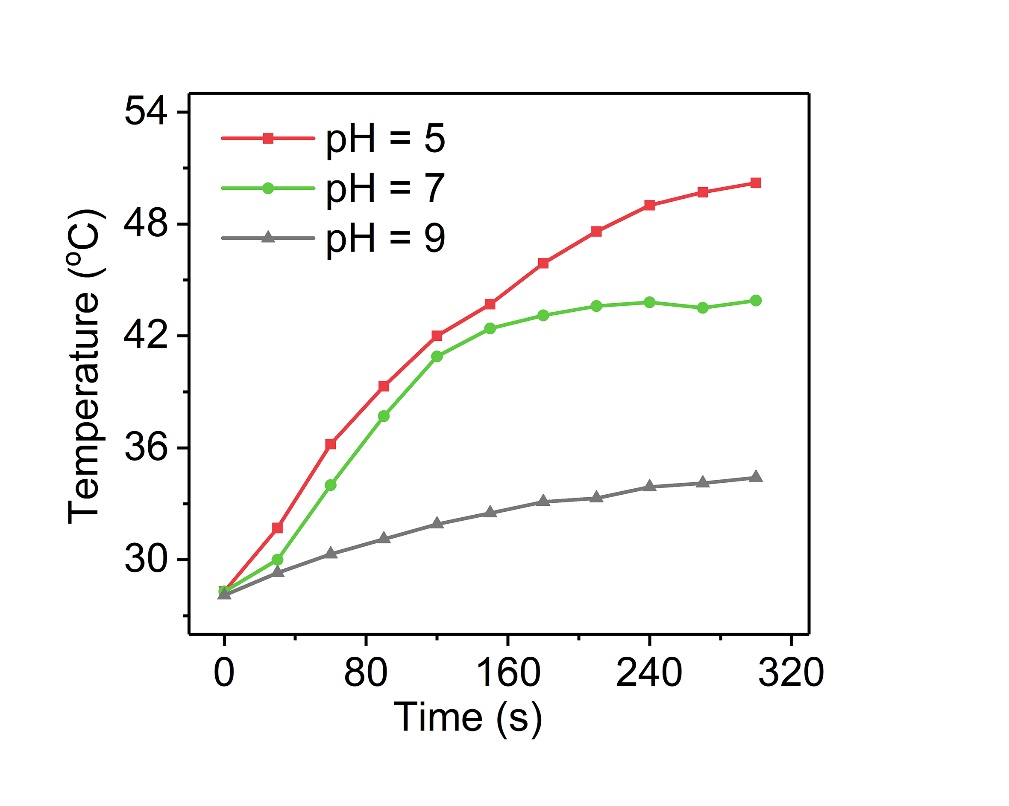


**Supplementary Figure 4.** Temperature changes of CTN solutions at pH = 5, 7 or 9 under 1064 nm laser irradiation at the power density of 1 W/cm^2^ for different time.


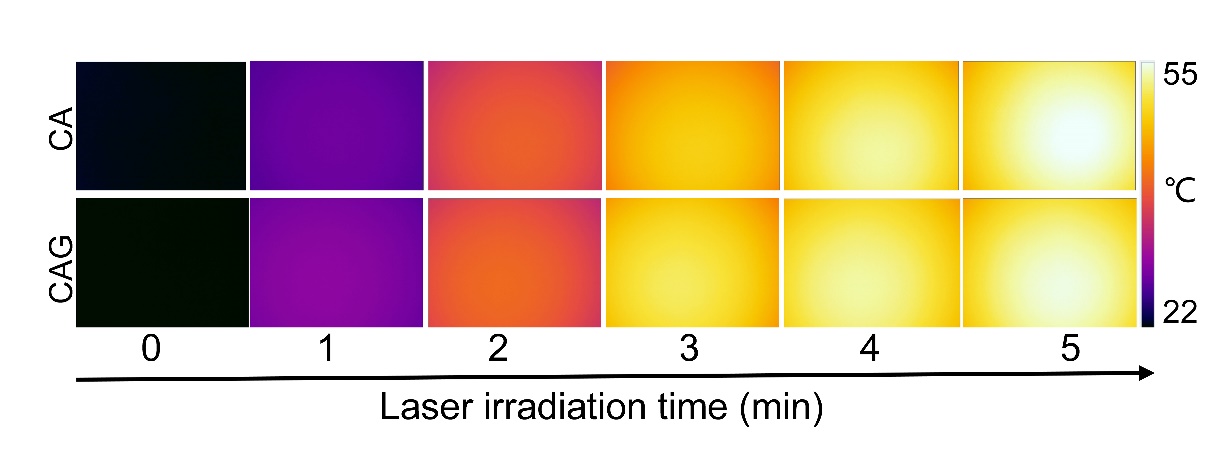


**Supplementary Figure 5.** Thermal images of solution containing CA or CAG hydrogels under 1064 nm laser irradiation at the power density of 1 W/cm^2^ for different time for 0, 1, 2, 3, 4, or 5 min.


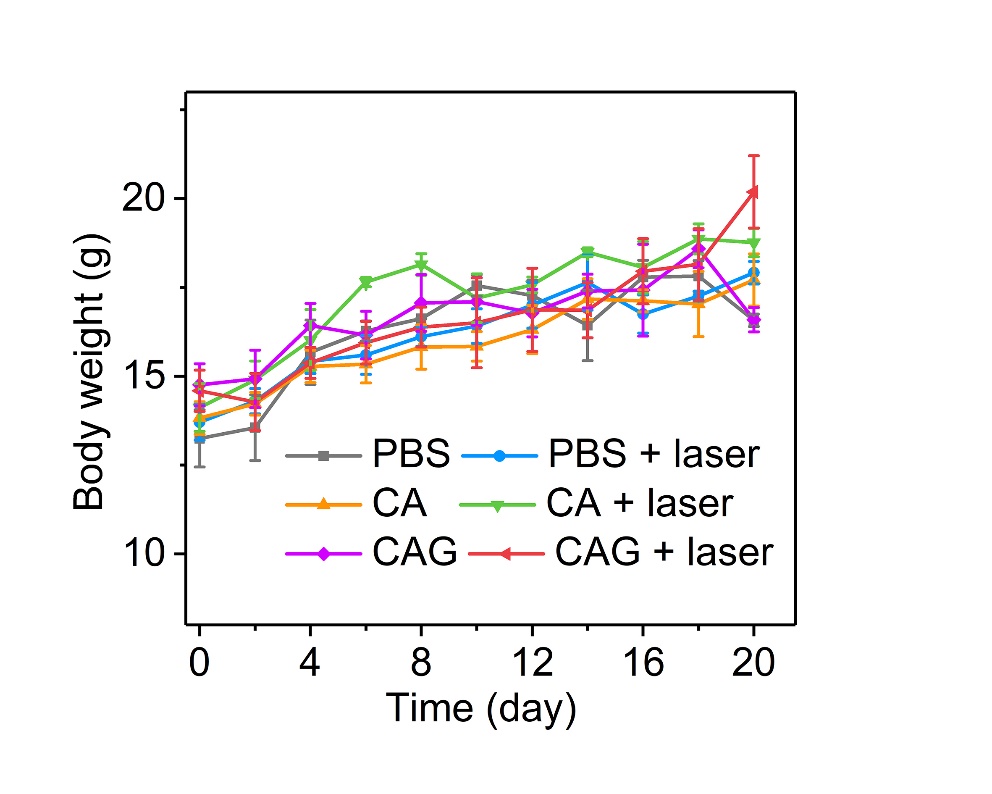


**Supplementary Figure 6.** Body weights of 4T1 tumor-bearing mice after treatment with PBS, CA, or CAG hydrogels without or with 1064 nm laser irradiation (1 W/cm^2^) for 10 min.


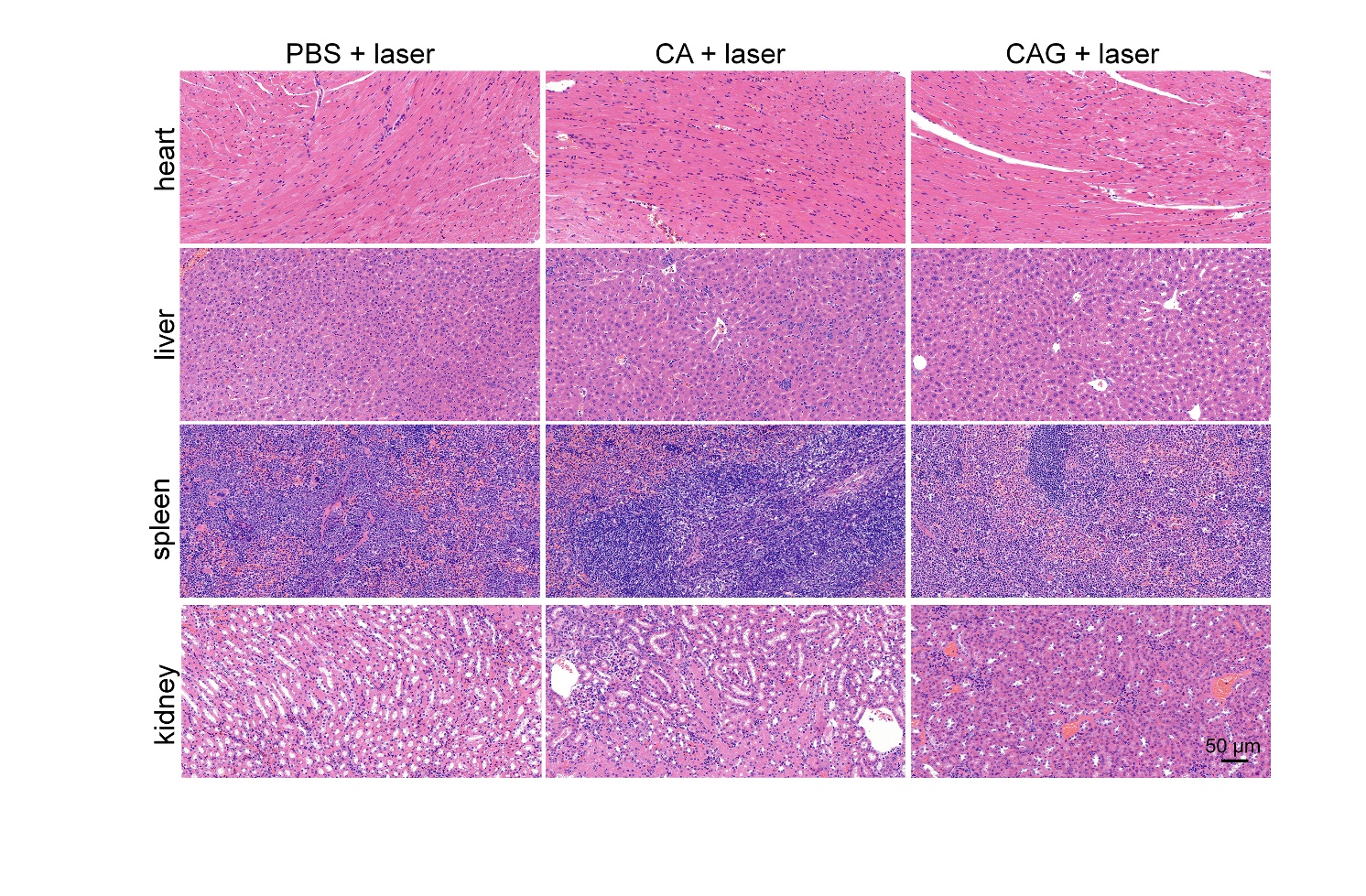


**Supplementary Figure 7.** H&E staining images of heart, liver, spleen, and kidney from 4T1 tumor-bearing mice after treatment with PBS, CA, or CAG hydrogels under 1064 nm laser irradiation (1 W/cm^2^) for 10 min.
